# PPI Network Analysis of mRNA Expression Profile of Ezrin Knockdown in Esophageal Squamous Cell Carcinoma

**DOI:** 10.1155/2014/651954

**Published:** 2014-07-14

**Authors:** Bingli Wu, Jianjun Xie, Zepeng Du, Jianyi Wu, Pixian Zhang, Liyan Xu, Enmin Li

**Affiliations:** ^1^Department of Biochemistry and Molecular Biology, Shantou University Medical College, Shantou 515041, China; ^2^Department of Pathology, Shantou Central Hospital, Shantou 515041, China; ^3^Institute of Oncologic Pathology, Shantou University Medical College, Shantou 515041, China

## Abstract

Ezrin, coding protein EZR which cross-links actin filaments, overexpresses and involves invasion, metastasis, and poor prognosis in various cancers including esophageal squamous cell carcinoma (ESCC). In our previous study, Ezrin was knock down and analyzed by mRNA expression profile which has not been fully mined. In this study, we applied protein-protein interactions (PPI) network knowledge and methods to explore our understanding of these differentially expressed genes (DEGs). PPI subnetworks showed that hundreds of DEGs interact with thousands of other proteins. Subcellular localization analyses found that the DEGs and their directly or indirectly interacting proteins distribute in multiple layers, which was applied to analyze the shortest paths between EZR and other DEGs. Gene ontology annotation generated a functional annotation map and found hundreds of significant terms, especially those associated with cytoskeleton organization of Ezrin protein, such as “cytoskeleton organization,” “regulation of actin filament-based process,” and “regulation of actin cytoskeleton organization.” The algorithm of Random Walk with Restart was applied to prioritize the DEGs and identified several cancer related DEGs ranked closest to EZR. These analyses based on PPI network have greatly expanded our comprehension of the mRNA expression profile of Ezrin knockdown for future examination of the roles and mechanisms of Ezrin.

## 1. Introduction

Ezrin (also named VIL2), which codes the protein EZR, is a member of the Ezrin-radixin-moesin (ERM) protein family that concentrates in actin rich cell-surface structures, cross-linking actin filaments with the plasma membrane [1]. It has been confirmed that Ezrin is overexpressed and involved various aspects of cancer cell biological behaviors, such as invasion and metastasis in breast cancer, osteosarcoma, and rhabdomyosarcoma. Moreover, the overexpression of Ezrin often correlates with poor prognosis of patients in cervical cancer, osteosarcoma, colorectal adenocarcinoma, and gastrointestinal cancers [2, 3].

The important biological role of Ezrin in human esophageal squamous cell carcinoma (ESCC) has been revealed in our previous studies. First, the overexpression of Ezrin is associated with the invasive phenotype of malignantly transformed esophageal epithelial cells [4]. We also found Ezrin protein EZR has a tendency to translocate from the plasma membrane to the cytoplasm in ESCC cells [5]. Subsequently, Ezrin was knock down by shRNA in ESCC cell line, which led to decrease of the growth, adhesion, and invasiveness of cancer cells* in vitro* and tumorigenesis* in vivo*. The mRNA expression profile of Ezrin knockdown was analyzed by Affymetrix GeneChip Human genome U133 plus 2.0 [6].

Various types of molecular interactions, such as protein-DNA, DNA-RNA, protein-RNA, RNA-RNA, and protein-protein interactions (PPI) play crucial roles in mediating numerous biological processes and endow the multifunctionality of a single protein. Most of proteins virtually form multiprotein complexes to achieve specific functions in the biological contexts [7, 8]. In recent years, an increasing emphasis has been put on integrated analysis of gene expression data in the context of PPI, which are widely applied in protein function prediction, functional modules identification, and interaction prediction [9, 10].

Nevertheless, the biological meaning of mRNA expression profile of Ezrin knockdown in ESCC has not been fully mined in our previous reports [6]. In this study, we reanalyzed the mRNA expression profile of Ezrin knockdown by integrating public PPI network to provide a deep view from a system level, which would be more comprehensive than merely listing the name of genes in the traditional way.

## 2. Materials and Methods

### 2.1. The Differentially Expressed Genes

GSE6233, the mRNA expression profile following Ezrin knockdown in the EC109 ESCC cell line, is available from GEO database (http://www.ncbi.nlm.nih.gov/geo/). The detailed microarray manipulation was described in our previous study [6]. Briefly, EC109 cells were transfected with pSUPER-siRNA specific for Ezrin, with pSUPER.neo vector of nonspecific siRNA used as a negative control. Stably transfected cell clones were selected by culture medium containing G418 (400 *μ*g/mL, Calbiochem, Germany). Total RNA of stable transfected clones was isolated by TRIzol (Invitrogen, USA) and was quantified spectrophotometrically. Double-stranded cDNA preparation, synthesis of biotin-labeled cRNA target, hybridization, washing and staining, subsequent scanning of the hybridized array, and data processing were performed according to the Manual of Affymetrix Gene Chip Expression Analysis Technical. The expression data was treated by normalization and log transform. The differentially expressed genes (DEGs) were obtained by the threshold of 2-fold change.

### 2.2. PPI Network Generation

The latest experimentally confirmed human PPI data is available from human protein-protein interactions database (HPRD) (http://www.hprd.org/), which has been widely applied in human PPI network research for various disease investigations [11]. The current HPRD PPI dataset contains 9617 unique proteins and 39140 edges (interactions). The HPRD data is loaded into Cytoscape software as a parent PPI network for subsequent new network construction [12].

First, three PPI subnetworks were generated by mapping the downregulated DEGs, upregulated DEGs, and total DEGs and extracting from the HPRD parent PPI network, respectively. To increase the reliability, the network reconstruction was limited to the first interacting protein neighbors of these DEGs. Second, to detect the axis of EZR-neighbors proteins-DEGs-neighbors proteins, EZR was used as query node to construct EZR-central PPI network. Third, a subnetwork was created by selecting nodes with all edges by Cytoscape after all DEGs were mapped to the HPRD PPI network to detect the internal interactions between DEGs. Single nodes and self-interactions of proteins in these subnetworks were removed.

### 2.3. Network Topological Parameters

There are several network topological parameters that enable the comparison and characterization of complex networks. The network topological parameters were analyzed by NetworkAnalyzer in this study [13]. Power law distribution of node degree, one of most important network topological characteristics, was analyzed as we performed previously [14].

### 2.4. PPI Network Subcellular Layer

The subcellular localization of each protein in the total DEGs PPI subnetwork was retrieved from the Uniprot protein database (http://www.hprd.org/) by a custom R program and was imported into Cytoscape as a node attribute. Cerebral (http://www.pathogenomics.ca/cerebral/) was applied for the protein layers according to their subcellular localization [15]. The total DEGs PPI subnetwork was divided into 8 layers according to their subcellular locations in this study as follows: Secreted, Secreted/Membrane, Membrane, Cytoskeleton/Cytoplasm, Cytoplasm, Cytoplasm/Nucleus, Nucleus, and Downstream genes. The proteins with unknown subcellular location were classified into Downstream genes. The igraph R program was applied to find the shortest path between EZR and ATF3 (activating transcription factor 3) in the total DEG PPI subnetwork. The protein members within these paths were also displayed according to their subcellular localization.

### 2.5. Construction of Functional Annotation Maps

To understand which aspects of biological function were involved for the proteins in the total DEG PPI subnetwork, ClueGO plugin was applied to identify the overrepresented “Biological Process” terms of gene ontology (GO) for protein members in the network. ClueGO integrates GO terms into PPI network and creates a functional annotation map indicating interrelations of terms [16]. A kappa score was calculated which reflects the relationships between the terms based on the similarity of their associated genes and we set the threshold as 0.3 in this study.

### 2.6. Random Walk with Restart to Prioritize DEGs

The algorithm of Random Walk with Restart (RWR) simulates a random walker on the network to compute the proximity between two nodes by exploiting the global structure of the network [17]. The algorithm calculates a priority score for each node based on the steady state probabilities. RWR is defined as the following equation:

(1) $$\begin{array}{c} p^{t+1}=\left(1-r\right)Wp^{t}+rp^{0}, \end{array}$$

where *r*  is the restart probability, *W* is the column-normalized adjacency matrix of the network graph, and *p* ^*t*^ is a vector of size equal to the number of nodes in the graph where the *i*th element holds the probability of being at node *i* at time step *t*. In this study, RWR was carried out by a custom R program in the total DEG PPI subnetwork with EZR protein set as the seed node (see Supplementary Material 1 available online at http://dx.doi.org/10.1155/2014/651954). We set the parameter *r* at 0.7, the same with Kohler's study [18]. And this parameter has been proved to have little influence to prioritization result in many other studies [19]. DEGs were ranked according to the values in the steady-state probability vector *P*_*∞*_. This was obtained at query time by performing the iteration until the change between *p* ^*t*^ and *p* ^*t*+1^ (measured by the *L*1 norm) fell below 10^−10^. The probabilities scores of DEGs were log 10 transformed and regarded as node attribute and displayed by Cytoscape.

## 3. Results

### 3.1. PPI Networks of DEGs

**Figure 1:**
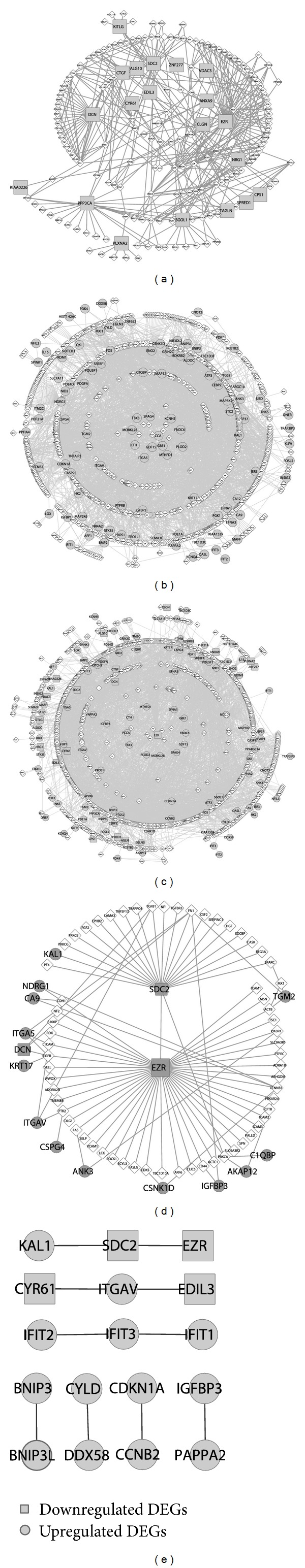
PPI subnetworks were constructed by mapping DEGs to HPRD PPI network. ((a)–(c)) PPI subnetworks for downregulated, upregulated, and total DEG, respectively. (d) EZR-central PPI subnetwork. (e) Internal interactions between DEGs. Square nodes represented proteins encoded by downregulated genes, while round nodes represented proteins encoded by upregulated genes. The other interacting proteins without significantly differentially expression were represented as diamond-shaped nodes.

Totally, we obtained 244 differentially expressed genes (DEGs), including 199 upregulated genes and 45 downregulated genes (Supplementary Material 2). It is critical to explore the potential roles of Ezrin by the explication of the DEGs; the investigation of their interactions with other proteins would provide a deep insight into the functions of Ezrin and its DEGs. The downregulated DEG PPI subnetwork contained 187 nodes and 384 edges (interactions), including 21 downregulated DEGs (Figure 1(a)). The upregulated DEG PPI subnetwork contained 799 nodes and 3097 edges, including 103 upregulated DEGs (Figure 1(b)). The total DEG PPI subnetwork composed of 942 nodes and 4095 edges, containing 123 DEGs (Figure 1(c)) (Supplementary Material 3). These three subnetworks indicated that knockdown of Ezrin greatly disturbed the PPI network in ESCC as hundreds of DEGs interact with thousands of proteins to enlarge the biological consequences. The subnetwork based on the axis of EZR-neighbors-DEGs-neighbors was also constructed to detect the relationship between EZR and its neatest DEG proteins (Figure 1(d)). This axis subnetwork composed of 79 nodes and 93 edges, including 16 DEGs; except for the downregulated EZR, SDC2, and DCN, the others were 13 upregulated genes. The current HPRD dataset contains 44 EZR interacting proteins, of these only SDC2 was downregulated 2.6-fold, while other proteins did not change significantly (Figure 1(d)). The DEG-DEG interactions were acquired and showed the internal interactions between DEGs. This subnetwork contained 17 nodes (4 downregulated nodes and 13 upregulated nodes) and 10 edges, forming 3 three-DEG interactions and 4 two-DEG interactions (Figure 1(e)). EZR connects with the upregulated KAL1 through its interaction with SDC2.

### 3.2. Analyses of Network Topological Properties

**Figure 2:**
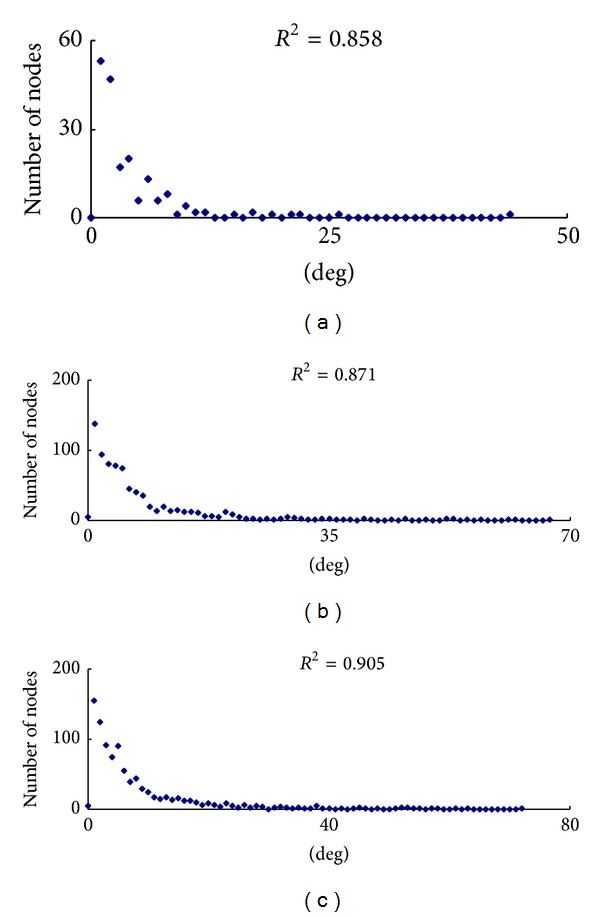
Power law distribution of node degree. (a) Degree distribution of the downregulated DEG PPI subnetwork. (b) Degree distribution of the upregulated DEG PPI subnetwork. (c) Degree distribution of the total DEG PPI subnetwork. The graph displays a decreasing trend of degree distribution with increase in number of links displaying scale-free topology.

Whether the node degree distribution of a network approximates a power law distribution is a standard character of scale-free networks. PPI network also obeys this rule, making it distinguished from random network [20]. The distributions of node degree of the downregulated, upregulated, and total DEG subnetworks approximately followed power law fit distributions, with an *R* ^2^ = 0.858, 0.871, and 0.905, respectively (Figure 2). These indicated three PPI subnetworks are true cellular complex biological networks characterized as scale-free. These results also suggest that a few protein nodes act as hubs with a large number of links to other protein nodes [21]. Other topological parameters of these subnetworks, such as clustering coefficient, network centralization, and network density were shown in Table 1. We also applied STRING database [22] to construct a new DEG PPI network (Supplementary Material 4), which is also characterized as scale-free (Supplementary Material 5). We compare several critical topological parameters of these two DEG PPI networks and find that these parameters are very similar (Supplementary Material 6). We consider our DEG PPI network derived from either HPRD or STRING is reliable and robust.

### 3.3. Subcellular Localization of Proteins in the PPI Subnetwork

**Figure 3:**
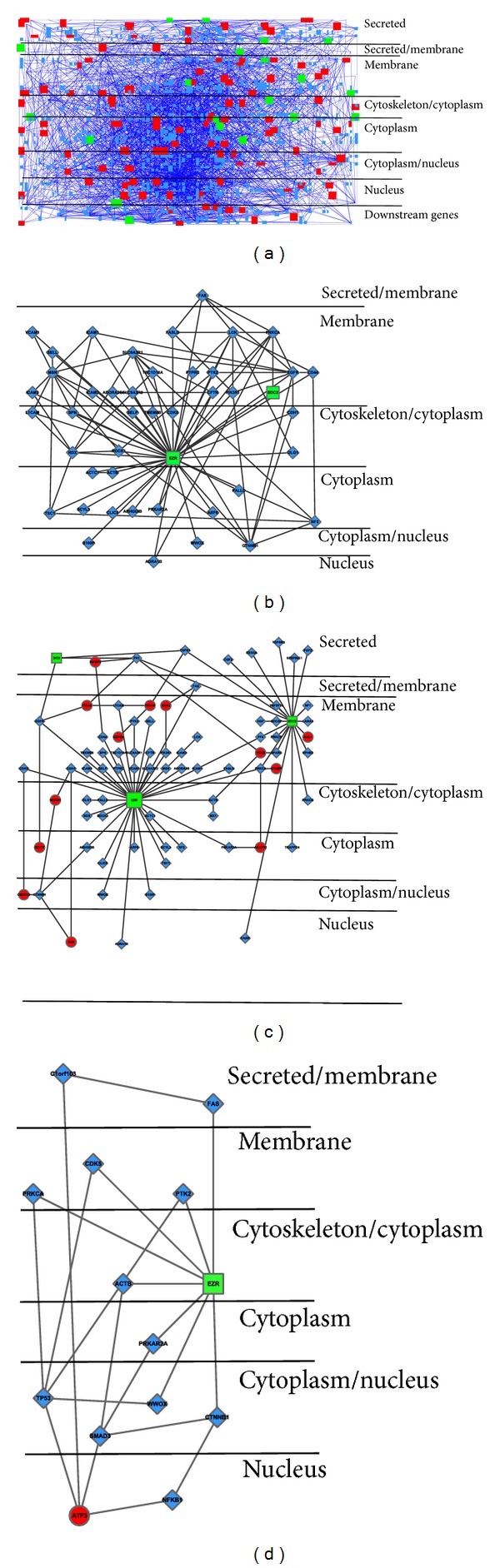
Subcellular localizations of proteins in the PPI subnetwork were illustrated by Cerebral. (a) The total DEG PPI subnetwork. (b) EZR and its interacting proteins. (c) EZR-central PPI subnetwork. (d) The shortest paths from EZR to ATF3.

After being synthesized, proteins are transported to cellular different compartments depending on their molecular roles, sometimes are even transported to multiple sites. Protein localization data is valuable information for elucidation of protein functions [23]. To show their subcellular localization and provide clues for their functions, the total DEG subnetwork was rearranged into 8 layers in this study (Figure 3(a)). As a linker between the actin cytoskeleton and plasma membrane proteins, Ezrin protein EZR mostly locates in the cytoskeleton/cytoplasm. Most of EZR interacting proteins mainly locate in membrane, cytoskeleton, or cytoplasm, where EZR mainly locates. However, four interacting proteins ADRA1B, S100P, WWOX, and CTNNB1 are able to translocate into nucleus (Figure 3(b)).

On the other hand, the proteins in the EZR-central network also involved various subcellular localizations (Figure 3(c)). Because a major component of signal flow in cellular signaling cascades is mediated by PPIs, we assumed various cellular signal transduction processes were built through the interactions between DEG and their neighbor proteins except the traditional acknowledged pathways. We have identified that transcription factor ATF3 was upregulated after Ezrin knockdown in our previous report [6]. To find the possible shortest path from EZR to ATF3, we applied the shortest path algorithm, which is able to find the shortest connection between two nodes in the graph, to identify the linking proteins between EZR and ATF3. We found nine shortest paths from EZR to ATF3 (Table 2) with all the lengths equal to 3.

For better illustration, these proteins were also rearranged into multiple layers according to their subcellular localizations (Figure 3(d)). Since most of the signal transduction is induced from cytoplasm to nucleus, we assumed the four following shortest paths had the maximum likelihood: EZR→ACTB→SMAD3→ATF3; EZR→PRKAR2A→SMAD3→ATF3; EZR→CTNNB1→NFKB1→ATF3; EZR→WWOX→TP53→ATF3.

### 3.4. Functional Annotation Map of PPI Subnetwork

**Figure 4:**
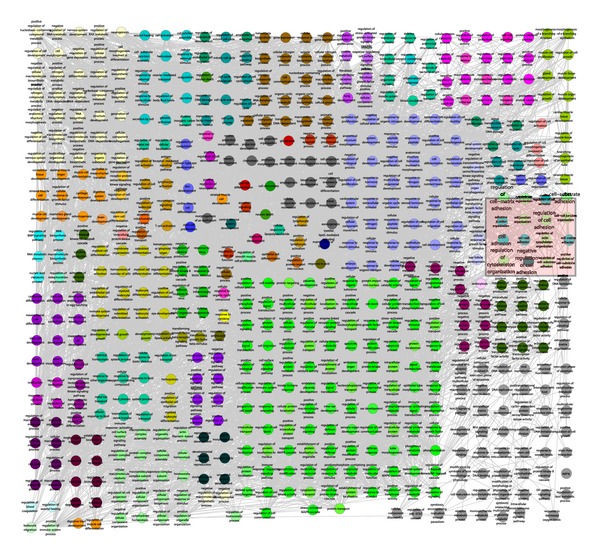
Functional annotation map of the total DEGs PPI subnetwork. The terms related to Ezrin functions were indicated by a pink shape.

ClueGO generated a functional annotation map for the total DEG PPI subnetwork, in which protein members were presented by nodes corresponding to their enriched GO terms, with edges indicating that two terms share the same enriched genes (Figure 4). Interestingly, many GO terms associated with cytoskeleton organization were found, such as “cytoskeleton organization,” “regulation of actin filament-based process,” and “regulation of actin cytoskeleton organization.” To our surprise, the PPI subnetwork also involved cell adhesion and extracellular matrix, such as “cell-cell junction organization,” “regulation of cell-matrix adhesion,” and “cell-substrate adhesion.” These results suggested that the knockdown of Ezrin affected various biological activities through the disturbed PPI subnetwork, which were closely consistent with the functions of EZR.

### 3.5. DEGs Prioritization

**Figure 5:**
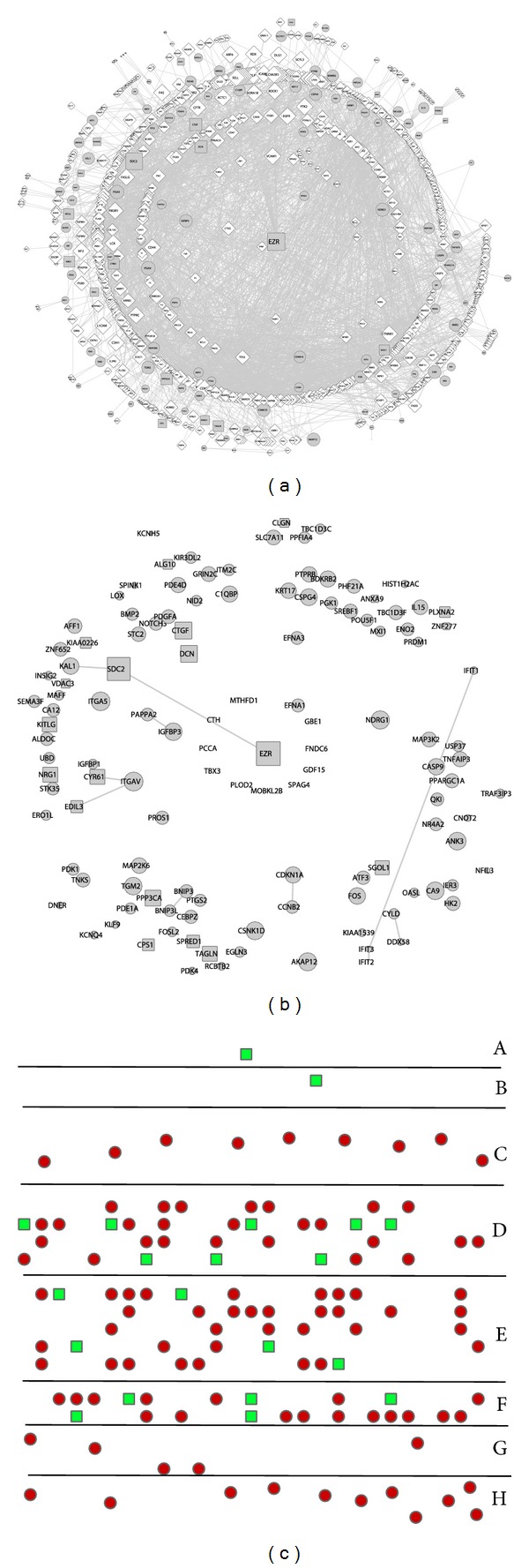
Prioritization analysis of DEGs in the total DEGs PPI subnetwork. (a) The size of each node in the PPI subnetwork was designed in a gradient based on the scores. (b) The DEGs were extracted from (a) to show their size. (c) The DEGs were rearranged according to their closeness to EZR protein.

Usually to obtain hundreds or thousands of DEGs from profile or -omics analyses, it is urgent to identify which DEGs are most related to the target gens(s) or which DEGs are expected to be investigated subsequently to reveal the underlie mechanisms. RWR algorithm was applied to prioritize the proteins in the total DEG PPI subnetwork with EZR set as seed node. The probability scores of DEGs ranged from −2.06 to −8.28 after log 10 transformation. The higher scores indicated the nodes were more closely connected with EZR. The scores were loaded as the node attributes of total PPI subnetwork by the indication of the node sizes (Figure 5(a)). The DEGs were solely displayed for a better distinguishing view (Figure 5(b)). To better illustrate their distance to EZR, the DEGs were classified according to the score range and rearranged into different layers; for example, EZR was classified as A, DEGs within score −2.0 ~ −2.99 were classified into B, DEGs within −3.0 ~ −3.99 were classified into C, and so on (Figure 5(c)). The downregulated SDC2 ranked the first closed DEGs to EZR, while other upregulated DEGs such as ITGA5 and NDRG1 were ranked the second class.

## 4. Discussion

ESCC is the fourth most frequently diagnosed cancer and the fourth leading cause of cancer death in China [24]. The biological roles and molecular mechanisms of Ezrin in ESCC are far from elaboration. A big challenge in the postgenomic era is to determine protein function at the scale level. Accumulated researches have demonstrated that an integrative analysis of gene expression profiles and PPI network can provide new lights into the molecular mechanisms of specific genes, or diseases [25, 26].

In this study, a system approach was developed by linking DEGs to public available PPI data to generate subnetworks, which provide unique insights into the mechanism of Ezrin from a network aspect. The three PPI subnetworks indicated EZR influent the protein activities through the directly or indirectly interactions with DEGs and other proteins, and its knockdown might affect various biological functions in ESCC. It has been suggested that methods based on network knowledge are important approaches for protein function annotation in the postgenomic era [27]. We would consider the downregulated EZR interacting protein SDC2 might also be crucial for the cytoskeleton organization before we began to search the literatures. Actually, Granés et al. reported that Ezrin links SDC2 to the actin cytoskeleton through the interaction between Ezrin N-terminal domain and SDC2 cytoplasmic domain, which confirmed our presumption [28].

The PPI subnetwork might provide clues to explain the potential molecular mechanisms that have not been revealed before. We previously confirmed that the knockdown of Ezrin decreased the invasion of ESCC cells through TGF-beta pathway with a decreased level of p-Smad2/3 [6]. However, the direct evidences have not been discovered. Recently, Mytilinaiou et al. demonstrated that the inhibition of SDC-2 abolished HT1080 cell adhesion through the inhibition of TGF-beta-induced Smad2 phosphorylation [29]. We found SDC2 was greatly decreased 2.6-fold in mRNA expression profile and confirmed the decrease of SDC2 as well as p-Smad2/3 in our previous report, which were consistent with the Mytilinaiou's report [6], while the downregulated CYR61 and CTGF are direct transcriptional targets of Smad2/3, with consensus Smad binding sequences in their promoters [30, 31]. By these combination analyses of PPI subnetwork and literatures, we suggested an axis of signal cascade of EZR (↓)→SDC2 (↓)→p-Smad2/3 (↓)→CYR61 and CTGF (↓).

Since the Ezrin knockdown induced a wide range change of gene expression profile, it is interesting to understand how this signal is transduced from cell front/surface into nucleus as EZR is a linker of membrane-cytoskeleton. One of the evidences is that the EZR directly interacting proteins, such as CTNNB1 (*β*-Catenin), S100P, WWOX, and ADRA1B, have the ability to translocate into nucleus [32–35]. Since so many directly and indirectly interacting proteins could translocate into nucleus, it is convinced that knockdown of Ezrin caused great impact on the ESCC gene expression profile. The localization of a protein is one of its most important attributes, which provides useful insight into the function of the protein and an in-depth understanding of how the biological processes are regulated by the intricate pathways [36]. In this study, subcellular localization information was incorporated into total DEG PPI subnetwork and generated more biologically intuitive pathway-like layouts of a network. These results indicated that EZR affected the signal cascades of extracellular-membrane-cytoskeleton/cytoplasm-nucleus. To illustrate the strength of this kind analysis, we applied shortest path algorithm to find the links between knockdown EZR and upregulated ATF3. Nine possible shortest paths were found. It was convinced that PPI network combined with protein subcellular localization provided great help in future experimental identification of the relationships between Ezrin and the DEGs.

How to choose critical gene from hundreds of DEGs is a big challenge for the researchers to continue the subsequent experiments after the chip experiment is finished. In this study, the algorithm RWR was applied to prioritize the DEGs by ranking their closeness to EZR. The most important advantage of RWR is that it can perform without any existing protein annotation, which is the limitation step for large scale protein analysis. Of the nearest DEGs closed to EZR, many of them are important for cytoskeleton organization and arrangement and are even involved in invasion and metastasis in multiple carcinomas. AKAP12, a scaffold protein for PKA and PKC, controls actin-cytoskeleton reorganization in a spatiotemporal manner [37]. ITGA5 is an important component for focal adhesions through a short cytoplasmic tail that structurally links the cytoskeleton to the extracellular matrix, transmitting mechanical signals across the plasma membrane in both directions and regulating cell migration [38]. These results provided the priorities of other DEGs by considering their relationship with EZR and provided important clues for experiments identification of DEGs.

Moreover, shortest path method has been widely used to discover disease genes on the network, such as colorectal cancer related genes and gastric cancer related genes [39, 40]. These two studies considered the proteins within the shortest path may share some common features of the two known cancer related genes and found dozens of new cancer related genes. In this research, we assumed that the information transmitted from our target protein EZR to another protein in the network would also adopt these most economic ways, so we applied the shortest path algorithm to illustrate how EZR reach a specific transcription factor. Similarly, we also found that some proteins in these shortest paths are ESCC related or at least cancer related. For example, the persistent SMAD3 phosphorylation is critical in the TGF-beta1-mediated EMT in ESCC [41]. In our previous study, we found that activating CTNNB1 (*β*-Catenin) signaling is important in the promotion of ESCC cell aggressiveness by downregulating DSC2 [42]. Inspired by the results described above, we also assumed the proteins close to EZR might also share some common features with EZR, suggesting these proteins might be very important in ESCC. The gene and protein levels of ITGA5 are increased in mucinous colorectal carcinomas [43]. Jin et al. found AKAP12 promoter hypermethylation can distinguish esophageal adenocarcinoma from esophageal squamous cell carcinoma and normal esophagus after they are detected in 259 human esophageal tissues [44]. So our both results of Random Walk with Restart and shortest path are different methods that are able to find key ESCC related genes.

## 5. Conclusion

In summary, the analyses based on PPI network have greatly expanded our understanding of the mRNA expression profile following Ezrin knockdown in ESCC. The GO annotation of PPI network provides a wide range of choice to explore the potential role of Ezrin. Both results from shortest paths and Random Walk with Restart analyses are able to find important ESCC related genes, which could serve as research targets in the future experiments to confirm the molecular mechanisms of Ezrin in ESCC.

## Supplementary Material

Supplementary Material 1: This is an R script for shortest path analysis using the igraph package.
Supplementary Material 2: Differentially expressed genes generated from the mRNA profile following Ezrin knockdown in esophageal squamous cell carcinoma using a 2-fold change threshold.
Supplementary Material 3: The detail protein-protein interaction pairs of the total differentially expressed gene PPI network.
Supplementary Material 4: A new DEG PPI network derived from STRING dataset, which contain 8362 nodes (179 DEGs) and 23117 edges. The black nodes present the DEGs, the white small nodes present their interacting proteins.
Supplementary Material 5: The distributions of node degree of new DEG network derived from STRING dataset.
Supplementary Material 6: Network topological parameters of the total DEG PPI sub-network derived from STRING.

## Figures and Tables

**Table 1:** Topological parameters of the downregulated, upregulated, and total DEGs PPI subnetwork.

PPI subnetwork	*y* = *βx* ^*a*^	*R* ^2^	Correlation	Clustering coefficient	Network centralization	Network density
Downregulated DEGs	*y* = 74.313*x* ^−1.357^	0.858	0.91	0.267	0.217	0.022
Upregulated DEGs	*y* = 452.48*x* ^−1.510^	0.871	0.839	0.163	0.074	0.01
Total DEGs	*y* = 631.42*x* ^−1.564^	0.905	0.823	0.16	0.068	0.009

**Table 2:** The nine shortest paths from EZR to ATF3.

Number	Protein members in the shortest paths
1	EZR→FAS→C1orf103→ATF3
2	EZR→CTNNB1→SMAD3→ATF3
3	EZR→ACTB→SMAD3→ATF3
4	EZR→PRKAR2A→SMAD3→ATF3
5	EZR→CTNNB1→NFKB1→ATF3
6	EZR→WWOX→TP53→ATF3
7	EZR→CDK5→TP53→ATF3
8	EZR→PRKCA→TP53→ATF3
9	EZR→PTK2→TP53→ATF3
