# Supplementary material for: PPI Network Analysis of mRNA Expression Profile of Ezrin Knockdown in Esophageal Squamous Cell Carcinoma
Source: Biomed Res Int. 2014 Jul 14;2014:651954. doi: 10.1155/2014/651954 (PMC4122099; doi:10.1155/2014/651954)
Supplement: Supplementary file 1 — Supplementary Material 1: This is an R script for shortest path analysis using the igraph package. Supplementary Material 2: Differentially expressed genes generated from the mRNA profile following Ezrin knockdown in esophageal squamous cell carcinoma using a 2-fold change threshold. Supplementary Material 3: The detail protein-protein interaction pairs of the total differentially expressed gene PPI network. Supplementary Material 4: A new DEG PPI network derived from STRING dataset, which contain 8362 nodes (179 DEGs) and 23117 edges. The black nodes present the DEGs, the white small nodes present their interacting proteins. Supplementary Material 5: The distributions of node degree of new DEG network derived from STRING dataset. Supplementary Material 6: Network topological parameters of the total DEG PPI sub-network derived from STRING. [file 651954.f1.pdf]

Supplementary Material I. Shortest path analysis R script

```
read.table("Ezrin siRNA PPI network.txt")->files
library(igraph)
graph.data.frame(files,directed=F)->PPIN
g1<-"EZR"
g2<-c("ATF3")
shortestpath<-get.all.shortest.paths(PPIN, g1, g2)
for(i in 1:length(shortestpath[[1]])){
print(V(PPIN)[shortestpath[[1]][[i]]])
}
```

Supplementary Material II. Differentially expressed genes from the mRNA profile following Ezrin knockdown in ESCC

Gene symbol	Official Full Name	Fold change
**Upregulated genes**		
IFI44	interferon-induced protein 44	14
C9ORF152	chromosome 9 open reading frame 152	10
WAPAL	wings apart-like homolog (Drosophila)	9.8
MGA	MAX gene associated	9.4
FEZF2	FEZ family zinc finger 2	9.2
OASL	2'-5'-oligoadenylate synthetase-like	8.4
GTDC1	glycosyltransferase-like domain containing 1	8
ATP13A5	ATPase type 13A5	7.8
LOC284561	uncharacterized LOC284561	7.6
AGAP1	ArfGAP with GTPase domain, ankyrin repeat and PH domain 1	7.4
MOBKL2B	MOB1, Mps One Binder kinase activator-like 2B (yeast)	7.2
PCCA	propionyl Coenzyme A carboxylase, alpha polypeptide	7.2
DDX58	DEAD (Asp-Glu-Ala-Asp) box polypeptide 58	7
NAPEPLD	N-acyl phosphatidylethanolamine phospholipase D	7
TM4SF4	transmembrane 4 L six family member 4	7
CARD16	caspase recruitment domain family, member 16	6.8
PLEKHH1	pleckstrin homology domain containing, family H (with MyTH4 domain) member 1	6.8
CSPG4	chondroitin sulfate proteoglycan 4	6.4
KIR3DL2	killer cell immunoglobulin-like receptor, three domains, long cytoplasmic tail, 2	6.4
LOC284408	hypothetical protein LOC338758	6.4
SEMA3F	sema domain, immunoglobulin domain (Ig), short basic domain, secreted, (semaphorin) 3F	6.4
C17ORF77	chromosome 17 open reading frame 77	6.2
GRIN2C	glutamate receptor, ionotropic, N-methyl D-aspartate 2C	6
OTUD1	OTU domain containing 1	6
ATAD2B	ATPase family, AAA domain containing 2B	5.6
C14ORF182	chromosome 14 open reading frame 182	5.6
STC1	stanniocalcin 1	5.6
ASMT	acetylserotonin O-methyltransferase	5.4
PTGS2	prostaglandin-endoperoxide synthase 2 (prostaglandin G/H synthase and cyclooxygenase)	5.4
QKI	quaking homolog, KH domain RNA binding (mouse)	5.4
OSTBETA	organic solute transporter beta	5.2
STK35	serine/threonine kinase 35	5.2
KCNQ4	potassium voltage-gated channel, KQT-like subfamily, member 4	5
TNKS	tankyrase, TRF1-interacting ankyrin-related ADP-ribose	5

	polymerase	
C7ORF60	chromosome 7 open reading frame 60	4.8
CLEC2B	C-type lectin domain family 2, member B	4.8
TBC1D3C	TTBC1 domain family, member 3C	4.8
TBC1D3F	TBC1 domain family, member 3F	4.8
DDX60	DEAD (Asp-Glu-Ala-Asp) box polypeptide 60	4.6
HSPC047	HSPC047	4.6
C1QBP	complement component 1, q subcomponent binding protein	4.2
FLJ10213	hypothetical protein FLJ10213	4.2
ISG15	ISG15 ubiquitin-like modifier	4.2
IFIT2	interferon-induced protein with tetratricopeptide repeats 2	4
UBD	ubiquitin D	4
BMP2	bone morphogenetic protein 2	3.8
H6PD	hexose-6-phosphate dehydrogenase (glucose 1-dehydrogenase)	3.8
RWDD2A	RWD domain containing 2A	3.8
SPINK6	serine peptidase inhibitor, Kazal type 6	3.8
VWA5B2	von Willebrand factor A domain containing 5B2	3.8
ZNF93	zinc finger protein 93	3.8
PFKFB4	6-phosphofructo-2-kinase/fructose-2,6-biphosphatase 4	3.6
TRAF3IP3	TRAF3 interacting protein 3	3.6
CSNK1D	casein kinase 1, delta	3.4
EGLN3	egl nine homolog 3 (C. elegans)	3.4
FAM13A	family with sequence similarity 13, member A	3.4
KCNH5	potassium voltage-gated channel, subfamily H (eag-related), member 5	3.4
POU5F1	POU class 5 homeobox 1	3.4
CA9	carbonic anhydrase IX	3.2
DHRS3	dehydrogenase/reductase (SDR family) member 3	3.2
IFIT1	interferon-induced protein with tetratricopeptide repeats 1	3.2
KAL1	Kallmann syndrome 1 sequence	3.2
MAP2K6	mitogen-activated protein kinase kinase 6	3.2
SPAG4	sperm associated antigen 4	3.2
C5ORF41	chromosome 5 open reading frame 41	3
GPR126	G protein-coupled receptor 126	3
IGFBP3	insulin-like growth factor binding protein 3	3
IL15	interleukin 15	3
LOX	lysyl oxidase	3
MTHFD1	methylenetetrahydrofolate dehydrogenase (NADP+ dependent) 1	3
NDRG1	N-myc downstream regulated 1	3
NFIL3	nuclear factor, interleukin 3 regulated	3
PRDM1	PR domain containing 1, with ZNF domain	3
SLCO1B3	solute carrier organic anion transporter family, member 1B3	3
TMEM45A	transmembrane protein 45A	3

BDKRB2	bradykinin receptor B2	2.8
BHLHE40	basic helix-loop-helix family, member e40	2.8
CNOT2	CCR4-NOT transcription complex, subunit 2	2.8
CYLD	cylindromatosis (turban tumor syndrome)	2.8
FOS	v-fos FBJ murine osteosarcoma viral oncogene homolog	2.8
ISG20	interferon stimulated exonuclease gene 20kDa	2.8
MAFF	v-maf musculoaponeurotic fibrosarcoma oncogene homolog F (avian)	2.8
NR4A2	nuclear receptor subfamily 4, group A, member 2	2.8
PDK1	pyruvate dehydrogenase kinase, isozyme 1	2.8
PPFIA4	protein tyrosine phosphatase, receptor type, f polypeptide (PTPRF), interacting protein (liprin), alpha 4	2.8
PROS1	protein S (alpha)	2.8
SORBS2	sorbin and SH3 domain containing 2	2.8
AKAP12	A kinase (PRKA) anchor protein 12	2.6
ANKZF1	ankyrin repeat and zinc finger domain containing 1	2.6
CTH	cystathionase (cystathionine gamma-lyase)	2.6
EFNA1	ephrin-A1	2.6
FOSL2	FOS-like antigen 2	2.6
FZD10	frizzled homolog 10 (Drosophila)	2.6
KIAA1539	KIAA1539	2.6
MXI1	MAX interactor 1	2.6
NEAT1	non-protein coding RNA 84	2.6
PDE4D	phosphodiesterase 4D	2.6
PTPRB	protein tyrosine phosphatase, receptor type, B	2.6
SNED1	sushi, nidogen and EGF-like domains 1	2.6
STC2	stanniocalcin 2	2.6
TBX3	T-box 3	2.6
ABCA5	ATP-binding cassette, sub-family A (ABC1), member 5	2.4
AK3L1	adenylate kinase 3-like 2; adenylate kinase 3-like 1	2.4
BNIP3	BCL2/adenovirus E1B 19kDa interacting protein 3	2.4
BNIP3L	BCL2/adenovirus E1B 19kDa interacting protein 3-like	2.4
C4ORF3	chromosome 4 open reading frame 3	2.4
CA12	carbonic anhydrase XII	2.4
CEBPZ	CCAAT/enhancer binding protein (C/EBP), zeta	2.4
CHST2	carbohydrate (N-acetylglucosamine-6-O) sulfotransferase 2	2.4
DEPDC6	DEP domain containing 6	2.4
ENO2	enolase 2 (gamma, neuronal)	2.4
FLCN	folliculin	2.4
HK2	hexokinase 2 pseudogene; hexokinase 2	2.4
IL20RB	interleukin 20 receptor beta	2.4
KLF9	Kruppel-like factor 9	2.4
LOC338758	hypothetisches Protein LOC338758	2.4
LOXL2	lysyl oxidase-like 2	2.4

NID2	nidogen 2 (osteonidogen)	2.4
PAPPA2	pappalysin 2	2.4
PDE1A	phosphodiesterase 1A, calmodulin-dependent	2.4
PLOD2	procollagen-lysine, 2-oxoglutarate 5-dioxygenase 2	2.4
PPP1R3B	protein phosphatase 1, regulatory (inhibitor) subunit 3B	2.4
QSOX1	quiescin Q6 sulfhydryl oxidase 1	2.4
SLC6A6	solute carrier family 6 (neurotransmitter transporter, taurine), member 6	2.4
SPINK1	serine peptidase inhibitor, Kazal type 1	2.4
TMEM27	transmembrane protein 27	2.4
TNFAIP3	tumor necrosis factor, alpha-induced protein 3	2.4
UBE2O	ubiquitin-conjugating enzyme E2O	2.4
WIPI1	WD repeat domain, phosphoinositide interacting 1	2.4
ZNF292	zinc finger protein 292	2.4
ZNF395	zinc finger protein 395	2.4
ATF3	activating transcription factor 3	2.2
ATP1B3	ATPase, Na+/K+ transporting, beta 3 polypeptide	2.2
CASP9	caspase 9, apoptosis-related cysteine peptidase	2.2
CDKN1A	cyclin-dependent kinase inhibitor 1A (p21, Cip1)	2.2
DNER	delta/notch-like EGF repeat containing	2.2
FOXN3	forkhead box N3	2.2
FUT11	fucosyltransferase 11 (alpha (1,3) fucosyltransferase)	2.2
GDF15	growth differentiation factor 15	2.2
HIST1H2BC	histone cluster 1, H2bc	2.2
IER3	immediate early response 3	2.2
IGFBP1	insulin-like growth factor binding protein 1	2.2
INSIG2	insulin induced gene 2	2.2
IQSEC2	IQ motif and Sec7 domain 2	2.2
ITGA5	integrin, alpha 5 (fibronectin receptor, alpha polypeptide)	2.2
ITM2C	integral membrane protein 2C	2.2
KDM3A	lysine (K)-specific demethylase 3A	2.2
KRT17	keratin 17; keratin 17 pseudogene 3	2.2
LOC285831	hypothetical protein LOC285831	2.2
PDK4	pyruvate dehydrogenase kinase, isozyme 4	2.2
PLEKHG1	pleckstrin homology domain containing, family G (with RhoGef domain) member 1	2.2
RCBTB2	regulator of chromosome condensation (RCC1) and BTB (POZ) domain containing protein 2	2.2
SLC2A13	solute carrier family 2 (facilitated glucose transporter), member 13	2.2
SLC7A11	solute carrier family 7, (cationic amino acid transporter, y+ system) member 11	2.2
SMOX	spermine oxidase	2.2
TMIE	transmembrane inner ear	2.2

WSB1	WD repeat and SOCS box-containing 1	2.2
ZDHHC11	zinc finger, DHHC-type containing 11	2.2
ZNF652	zinc finger protein 652	2.2
AFF1	AF4/FMR2 family, member 1	2
ALDOC	aldolase C, fructose-bisphosphate	2
ANK3	ankyrin 3, node of Ranvier (ankyrin G)	2
ANKRD37	ankyrin repeat domain 37	2
C11ORF86	chromosome 11 open reading frame 86	2
C22ORF31	chromosome 22 open reading frame 31	2
CCNB2	cyclin B2	2
CD83	CD83 molecule	2
EFNA3	ephrin-A3	2
ERO1L	ERO1-like (S. cerevisiae)	2
FAM117B	family with sequence similarity 117, member B	2
GBE1	glucan (1,4-alpha-), branching enzyme 1	2
HILPDA	histone cluster 1, H2ac	2
HIST1H2AC	histone cluster 1, H2ac	2
HIST1H2AG	histone cluster 1, H2ag	2
HIST2H2AA3	histone cluster 2, H2aa3; histone cluster 2, H2aa4	2
IFIT3	interferon-induced protein with tetratricopeptide repeats 3	2
ITGAV	integrin, alpha V (vitronectin receptor, alpha polypeptide, antigen CD51)	2
KYNU	kynureninase (L-kynurenine hydrolase)	2
MAP3K2	mitogen-activated protein kinase kinase kinase 2	2
NAPB	N-ethylmaleimide-sensitive factor attachment protein, beta	2
NOTCH3	Notch homolog 3 (Drosophila)	2
PABPC4L	poly(A) binding protein, cytoplasmic 4-like	2
PDGFA	platelet-derived growth factor alpha polypeptide	2
PGK1	phosphoglycerate kinase 1	2
PHF21A	PHD finger protein 21A	2
PPARGC1A	peroxisome proliferator-activated receptor gamma, coactivator 1 alpha	2
RASSF9	Ras association (RalGDS/AF-6) domain family (N-terminal) member 9	2
SLC16A6	solute carrier family 16, member 6 (monocarboxylic acid transporter 7); similar to solute carrier family 16, member 6	2
SLC17A5	solute carrier family 17 (anion/sugar transporter), member 5	2
SLC7A1	solute carrier family 7 (cationic amino acid transporter, y+ system), member 1	2
SMOC1	SPARC related modular calcium binding 1	2
SNX14	sorting nexin 14	2
SREBF1	sterol regulatory element binding transcription factor 1	2
SYT12	synaptotagmin XII	2
TANC2	tetratricopeptide repeat, ankyrin repeat and coiled-coil	2

	containing 2	
TGM2	transglutaminase 2 (C polypeptide, protein-glutamine-gamma-glutamyltransferase)	2
TMEM47	transmembrane protein 47	2
USP37	ubiquitin specific peptidase 37	2
ZNF48	zinc finger protein 48	2
**Downregulated genes**		
ABCA13	ATP-binding cassette, sub-family A (ABC1), member 13	-2
C14ORF126	chromosome 14 open reading frame 126	-2
CYR61	cysteine-rich, angiogenic inducer, 61	-2
DHDPSL	N-acetylneuraminate pyruvate lyase 2 (putative)	-2
GPR56	G protein-coupled receptor 56	-2
KLHDC7A	kelch domain containing 7A	-2
LOC133926	hypothetical protein LOC133926	-2
NRG1	neuregulin 1	-2
KITLG	KIT ligand	-2.2
CXORF48	similar to chromosome X open reading frame 48; chromosome X open reading frame 48	-2.4
MDFIC	MyoD family inhibitor domain containing	-2.4
MPP7	membrane protein, palmitoylated 7 (MAGUK p55 subfamily member 7)	-2.4
EDIL3	EGF-like repeats and discoidin I-like domains 3	-2.6
PPP3CA	protein phosphatase 3 (formerly 2B), catalytic subunit, alpha isoform	-2.6
SDC2	syndecan 2	-2.6
CYB561	cytochrome b-561	-2.8
OLFML2B	olfactomedin-like 2B	-2.8
MUC12	mucin 12, cell surface associated; similar to mucin 11	-3
OLFML1	olfactomedin-like 1	-3
MT1E	metallothionein 1E	-3.2
CTGF	connective tissue growth factor	-3.6
TAGLN	transgelin	-3.6
ALG10	asparagine-linked glycosylation 10, alpha-1,2-glucosyltransferase homolog (S. pombe)	-4
ANXA9	annexin A9	-4
SPRED1	sprouty-related, EVH1 domain containing 1	-4.2
DCN	decorin	-4.4
PAGE1	P antigen family, member 1 (prostate associated)	-5.2
FAM179A	family with sequence similarity 179, member A	-5.4
HEATR7B2	HEAT repeat family member 7B2	-5.4
ZNF277	zinc finger protein 277	-5.4
ZNF610	zinc finger protein 610	-5.4
PLXNA2	plexin A2	-5.6
CPS1	carbamoyl-phosphate synthetase 1, mitochondrial	-6.2

VDAC3	voltage-dependent anion channel 3	-6.2
KRT4	keratin 4	-6.4
MUC12	mucin 12, cell surface associated; similar to mucin 11	-6.4
DPY19L2	dpy-19-like 2 (C. elegans)	-6.6
ARL4D	ADP-ribosylation factor-like 4D	-7
KIAA0226	KIAA0226	-7
CLGN	calmegin	-7.6
HSPA9	heat shock 70kDa protein 9 (mortalin)	-7.6
NCRNA00032	non-protein coding RNA 32	-8.4
C6ORF162	chromosome 6 open reading frame 162	-8.6
A2BP1	ataxin 2-binding protein 1	-9
SGOL1	shugoshin-like 1 (S. pombe)	-11

Supplementary Material III. Ezrin siRNA PPI network

ADAM19	pp	A2M
HSPA5	pp	A2M
KLK3	pp	A2M
LRP1	pp	A2M
MMP2	pp	A2M
PDGFA	pp	A2M
ABL1	pp	ABL1
BCAR1	pp	ABL1
CDK1	pp	ABL1
EPHB2	pp	ABL1
KIT	pp	ABL1
PRKDC	pp	ABL1
TP53	pp	ABL1
BDKRB2	pp	ACE
NDRG1	pp	ACSL3
ABL1	pp	ACTA1
ACTA1	pp	ACTA1
CLIC5	pp	ACTA1
DLG1	pp	ACTA1
EGFR	pp	ACTA1
HCLS1	pp	ACTA1
PRKCA	pp	ACTA1
PRKCD	pp	ACTA1
PRKCE	pp	ACTA1
RAC1	pp	ACTA1
TAGLN	pp	ACTA1
TMSB4X	pp	ACTA1
TP53	pp	ACTA1
ACTB	pp	ACTB
ACTG1	pp	ACTB
EZR	pp	ACTB
GAPDH	pp	ACTB
HSD17B4	pp	ACTB
LMNA	pp	ACTB
LYN	pp	ACTB
PLD2	pp	ACTB
PRKCD	pp	ACTB
PTN	pp	ACTB
RAC1	pp	ACTB
SMAD3	pp	ACTB
TGM2	pp	ACTB
TMSB4X	pp	ACTB
ACTB	pp	ACTC1

EZR	pp	ACTC1
PRKCE	pp	ACTC1
ACTG1	pp	ACTG1
NDRG1	pp	ACTG1
SUMO4	pp	ACTG1
TMSB4X	pp	ACTG1
BMP2	pp	ACVR1
ENG	pp	ACVR1
SMAD9	pp	ACVR1
BMP2	pp	ACVR2B
EPHA3	pp	ADAM10
FURIN	pp	ADAM19
CLGN	pp	ADAM2
AP2M1	pp	ADRA1B
EZR	pp	ADRA1B
GNA11	pp	ADRA1B
NOS1	pp	ADRA1B
PRKCA	pp	ADRA1B
STAT1	pp	ADRA1B
AKAP12	pp	ADRB2
AKAP5	pp	ADRB2
ARRB2	pp	ADRB2
GNA11	pp	ADRB2
GRK5	pp	ADRB2
GRK6	pp	ADRB2
NOS1	pp	ADRB2
PRKCD	pp	ADRB2
SLC9A3R1	pp	ADRB2
SLC9A3R2	pp	ADRB2
AGTR1	pp	ADRBK1
AKAP12	pp	ADRBK1
CAPN2	pp	ADRBK1
CAV1	pp	ADRBK1
EGFR	pp	ADRBK1
GIT1	pp	ADRBK1
MAPK3	pp	ADRBK1
PRKACA	pp	ADRBK1
PRKCA	pp	ADRBK1
PRKCB	pp	ADRBK1
PRKCD	pp	ADRBK1
PRKCG	pp	ADRBK1
AGTR1	pp	ADRBK2
GIT1	pp	ADRBK2
PRDM1	pp	AES

MLLT1	pp	AFF1
PPFIA4	pp	AGTPBP1
ARRB2	pp	AGTR1
BDKRB2	pp	AGTR1
NOS3	pp	AGTR1
RAB5A	pp	AGTR1
STC2	pp	AIFM1
GABRB1	pp	AKAP5
FNBP1	pp	AKAP9
PKN1	pp	AKAP9
PPP1CA	pp	AKAP9
AKT1	pp	AKT1
BCL10	pp	AKT1
CASP3	pp	AKT1
ILK	pp	AKT1
PKN2	pp	AKT1
PPP2CA	pp	AKT1
PRKCZ	pp	AKT1
RPS6KB1	pp	AKT1
TSC1	pp	AKT1
VEGFA	pp	AKT1
ALDOA	pp	ALDOA
PLD2	pp	ALDOA
SUMO4	pp	ALDOA
TGM2	pp	ALDOA
TUBB	pp	ALDOA
PLD2	pp	ALDOC
ANGPT1	pp	ANGPT1
KCNC1	pp	ANK3
SCN2A	pp	ANK3
SCNN1B	pp	ANK3
SMAD2	pp	ANK3
SMAD3	pp	ANK3
SPTBN4	pp	ANK3
TGM2	pp	ANXA1
AP1G1	pp	AP1G1
AP1M2	pp	AP1G1
NDRG1	pp	AP1M2
AP2M1	pp	AP2M1
ARRB2	pp	AP2M1
NDRG1	pp	AP2M1
CTGF	pp	AP3D1
CASP9	pp	APAF1
FAS	pp	APAF1

HIP1	pp	APAF1
PPP1CA	pp	APAF1
APOA1	pp	APOA1
AES	pp	AR
AKT1	pp	AR
AR	pp	AR
ATF2	pp	AR
CALM1	pp	AR
CAV1	pp	AR
CDK1	pp	AR
CREBBP	pp	AR
EGFR	pp	AR
HDAC1	pp	AR
HIF1A	pp	AR
KAT5	pp	AR
MAPK1	pp	AR
MED1	pp	AR
MYBBP1A	pp	AR
NCOA1	pp	AR
NISCH	pp	AR
NR0B1	pp	AR
PIAS4	pp	AR
POU5F1	pp	AR
RUNX1	pp	AR
SMAD3	pp	AR
SMAD4	pp	AR
SP1	pp	AR
SUMO1	pp	AR
TSG101	pp	AR
UBE2I	pp	AR
ARAF	pp	ARAF
MAP2K2	pp	ARAF
YWHAG	pp	ARAF
AP3D1	pp	ARF6
RHOA	pp	ARHGAP5
CASP3	pp	ARHGDIB
ANXA9	pp	ARHGEF10L
SCN2A	pp	ARHGEF10L
TLE1	pp	ARL4D
ARF6	pp	ARRB2
CLTC	pp	ARRB2
CSNK2A1	pp	ARRB2
CSNK2A2	pp	ARRB2
FLNA	pp	ARRB2

MAP3K5	pp	ARRB2
MAPK1	pp	ARRB2
RAF1	pp	ARRB2
TGFBR3	pp	ARRB2
ASCL1	pp	ASCL1
BMP2	pp	ASCL1
UBQLN1	pp	ASCL1
ATF2	pp	ATF2
CEBPG	pp	ATF2
FOS	pp	ATF2
GTF2F2	pp	ATF2
JUN	pp	ATF2
MAPK13	pp	ATF2
MAPK14	pp	ATF2
MAPK8	pp	ATF2
RB1	pp	ATF2
RUVBL2	pp	ATF2
SMAD3	pp	ATF2
SMAD4	pp	ATF2
UBE2I	pp	ATF2
ATF3	pp	ATF3
BATF3	pp	ATF3
ATF2	pp	ATF7
A2M	pp	ATF7IP
ACTB	pp	ATF7IP
ACTG1	pp	ATF7IP
CSPG4	pp	ATF7IP
FLT4	pp	ATF7IP
GMEB1	pp	ATF7IP
HSPG2	pp	ATF7IP
LAMC1	pp	ATF7IP
PAPPA2	pp	ATF7IP
POLR2C	pp	ATF7IP
EGFR	pp	ATP1A1
MAPK3	pp	ATP1A1
NDRG1	pp	ATP1A1
CASK	pp	ATP2B4
DLG1	pp	ATP2B4
DLG3	pp	ATP2B4
DLG4	pp	ATP2B4
NOS1	pp	ATP2B4
AKT1	pp	ATXN1
ATXN1	pp	ATXN1
CDK6	pp	ATXN1

PRDM1	pp	ATXN1
PSMC3	pp	ATXN1
PTGDS	pp	ATXN1
UBQLN4	pp	ATXN1
YWHAE	pp	ATXN1
AZGP1	pp	AZGP1
SLC7A11	pp	BAAT
CSNK1D	pp	BACE1
FURIN	pp	BACE1
ITM2C	pp	BACE1
AKT1	pp	BAD
EWSR1	pp	BAD
HRK	pp	BAD
MAPK8	pp	BAD
MCL1	pp	BAD
PIM1	pp	BAD
PPP1CA	pp	BAD
PPP3CA	pp	BAD
PRKACA	pp	BAD
RAF1	pp	BAD
RPS6KA1	pp	BAD
YWHAE	pp	BAD
ATXN1	pp	BAT2
C1QBP	pp	BAT2
EIF3E	pp	BAT2
PSMA3	pp	BAT2
UBAP2L	pp	BAT2
BATF	pp	BATF
DDIT3	pp	BATF
PGR	pp	BATF3
CASP3	pp	BCAR1
ESR1	pp	BCAR1
HSPA5	pp	BCAR1
PTK2	pp	BCAR1
TUBA4A	pp	BCAR1
YWHAZ	pp	BCAR1
PTN	pp	BCCIP
BCL10	pp	BCL10
MAP3K7	pp	BCL10
BAD	pp	BCL2
BCL2	pp	BCL2
BCL2L1	pp	BCL2
BNIP3	pp	BCL2
BNIP3L	pp	BCL2

CASP3	pp	BCL2
FKBP8	pp	BCL2
HRK	pp	BCL2
MAPK1	pp	BCL2
MAPK8	pp	BCL2
NLRP1	pp	BCL2
PARP1	pp	BCL2
PPP1CA	pp	BCL2
PPP2CA	pp	BCL2
PPP2R5A	pp	BCL2
PRKCA	pp	BCL2
RAF1	pp	BCL2
TP53	pp	BCL2
APAF1	pp	BCL2L1
BAD	pp	BCL2L1
BCL2L1	pp	BCL2L1
BNIP3	pp	BCL2L1
BNIP3L	pp	BCL2L1
CASP9	pp	BCL2L1
FKBP8	pp	BCL2L1
HRK	pp	BCL2L1
MCL1	pp	BCL2L1
NLRP1	pp	BCL2L1
PPP1CA	pp	BCL2L1
PSEN2	pp	BCL2L1
COPS5	pp	BCL3
CREBBP	pp	BCL3
FOS	pp	BCL3
JUN	pp	BCL3
KAT5	pp	BCL3
NCOA1	pp	BCL3
NFKB1	pp	BCL3
RXRA	pp	BCL3
TBP	pp	BCL3
ADRBK1	pp	BDKRB2
ADRBK2	pp	BDKRB2
GNA11	pp	BDKRB2
GRK4	pp	BDKRB2
GRK5	pp	BDKRB2
GRK6	pp	BDKRB2
NOS1	pp	BDKRB2
NOS3	pp	BDKRB2
PRKCD	pp	BDKRB2
PTPN11	pp	BDKRB2

DIABLO	pp	BIRC2
IKBKG	pp	BIRC2
TRAF2	pp	BIRC2
CASP9	pp	BIRC3
DIABLO	pp	BIRC3
TRAF2	pp	BIRC3
BIRC5	pp	BIRC5
BIRC6	pp	BIRC5
CDK1	pp	BIRC5
DIABLO	pp	BIRC5
HSP90AA1	pp	BIRC5
XIAP	pp	BIRC5
CASP9	pp	BIRC8
BMP2	pp	BMP2
BMPER	pp	BMP2
BMPR1A	pp	BMP2
BMPR1B	pp	BMP2
BMPR2	pp	BMP2
CHRDL2	pp	BMP2
COL2A1	pp	BMP2
ENG	pp	BMP2
GREM2	pp	BMP2
MGP	pp	BMP2
NOG	pp	BMP2
SOSTDC1	pp	BMP2
TGFB1	pp	BMP2
TGFB2	pp	BMP2
BMPR1A	pp	BMPR1A
BMPR1B	pp	BMPR1A
BMPR1B	pp	BMPR1B
TRAF6	pp	BMPR1B
BMPR1A	pp	BMPR2
BMPR1B	pp	BMPR2
BMPR2	pp	BMPR2
LIMK1	pp	BMPR2
BNIP3	pp	BNIP3
BNIP3	pp	BNIP3L
BNIP3L	pp	BNIP3L
EWSR1	pp	BNIP3L
STEAP3	pp	BNIP3L
QKI	pp	C10orf10
CA12	pp	C15orf29
ENO2	pp	C16orf45
PPP3CA	pp	C16orf74

ATF3	pp	C1orf103
C1orf103	pp	C1orf103
CDC42	pp	C1orf103
FAS	pp	C1orf103
HMGB1	pp	C1orf103
MLLT3	pp	C1orf103
MNAT1	pp	C1orf103
POLR2C	pp	C1orf103
POLR3F	pp	C1orf103
PRMT1	pp	C1orf103
PSMD2	pp	C1orf103
RPLP1	pp	C1orf103
TK1	pp	C1orf103
VIM	pp	C1orf103
CDKN1A	pp	C1orf123
C1QBP	pp	C1QA
CSPG4	pp	C1QA
DCN	pp	C1QA
FN1	pp	C1QA
C1QBP	pp	C1QBP
EXOSC6	pp	C1QBP
GAB1	pp	C1QBP
GABRB1	pp	C1QBP
HABP4	pp	C1QBP
HRK	pp	C1QBP
MAPK3	pp	C1QBP
MMP14	pp	C1QBP
NFYB	pp	C1QBP
PRKD1	pp	C1QBP
SFRS9	pp	C1QBP
TOP3B	pp	C1QBP
YWHAB	pp	C1QBP
YWHAG	pp	C1QBP
BMPR2	pp	C4BPA
PROS1	pp	C4BPA
ENO2	pp	C6orf47
CDKN1A	pp	C7orf20
EEF1G	pp	C7orf20
KAT5	pp	C7orf20
ATP5C1	pp	CA12
CA12	pp	CA12
CABIN1	pp	CABIN1
AKAP5	pp	CALM1
CABIN1	pp	CALM1

CALM1	pp	CALM1
CCND1	pp	CALM1
CCNE1	pp	CALM1
CSNK2A1	pp	CALM1
CSNK2A2	pp	CALM1
DDX5	pp	CALM1
DLG3	pp	CALM1
EGFR	pp	CALM1
ESR1	pp	CALM1
ESR2	pp	CALM1
ESRRG	pp	CALM1
EWSR1	pp	CALM1
FAS	pp	CALM1
GRK4	pp	CALM1
HSP90AA1	pp	CALM1
KCNQ3	pp	CALM1
NR3C1	pp	CALM1
PDE1A	pp	CALM1
PLCD1	pp	CALM1
RELA	pp	CALM1
SNCA	pp	CALM1
YWHAE	pp	CALM1
AR	pp	CALR
CALR	pp	CALR
F5	pp	CALR
F8	pp	CALR
FBN1	pp	CALR
ITGAV	pp	CALR
NR3C1	pp	CALR
PDIA3	pp	CALR
PLP1	pp	CALR
SUMO4	pp	CALR
TF	pp	CALR
THBS1	pp	CALR
CFTR	pp	CANX
F8	pp	CANX
HLA-A	pp	CANX
ITGB1	pp	CANX
MTNR1B	pp	CANX
NDRG1	pp	CANX
PDIA3	pp	CANX
PLP1	pp	CANX
TF	pp	CANX
CASP9	pp	CAPN2

CARD8	pp	CARD8
STC2	pp	CARHSP1
CASK	pp	CASK
DLG1	pp	CASK
DLG4	pp	CASK
F11R	pp	CASK
CASP10	pp	CASP10
CASP9	pp	CASP10
FADD	pp	CASP10
AIFM1	pp	CASP3
APAF1	pp	CASP3
AR	pp	CASP3
BIRC2	pp	CASP3
BIRC3	pp	CASP3
BIRC5	pp	CASP3
BIRC6	pp	CASP3
BIRC7	pp	CASP3
CASP10	pp	CASP3
CASP3	pp	CASP3
CASP9	pp	CASP3
DCTN1	pp	CASP3
GZMB	pp	CASP3
HCLS1	pp	CASP3
HIP1	pp	CASP3
MET	pp	CASP3
ABL1	pp	CASP9
AKT1	pp	CASP9
BCL10	pp	CASP9
BIRC2	pp	CASP9
BIRC5	pp	CASP9
BIRC6	pp	CASP9
BIRC7	pp	CASP9
CARD8	pp	CASP9
CASP9	pp	CASP9
GMEB1	pp	CASP9
GZMB	pp	CASP9
HIP1	pp	CASP9
ILK	pp	CASP9
LYN	pp	CASP9
MAP2K1	pp	CASP9
MAP2K2	pp	CASP9
MAPK1	pp	CASP9
MAPK3	pp	CASP9
NLRP1	pp	CASP9

NOD1	pp	CASP9
PRKCZ	pp	CASP9
SRF	pp	CASP9
CASP3	pp	CAST
ABL1	pp	CAV1
CAV1	pp	CAV1
CSNK2A1	pp	CAV1
CSNK2A2	pp	CAV1
EGFR	pp	CAV1
IGF1R	pp	CAV1
IGFBP3	pp	CAV1
LRP1	pp	CAV1
MAPK1	pp	CAV1
MMP14	pp	CAV1
PLD2	pp	CAV1
RAC1	pp	CAV1
TRAF2	pp	CAV1
CBFA2T3	pp	CBFA2T2
SIN3A	pp	CBFA2T2
ZNF652	pp	CBFA2T2
CBFA2T3	pp	CBFA2T3
ZNF652	pp	CBFA2T3
IFIT3	pp	CCDC67
C10orf10	pp	CCDC85B
CCDC67	pp	CCDC85B
CDKN1A	pp	CCDC85B
COPS4	pp	CCDC85B
HMG20B	pp	CCDC85B
KIAA1539	pp	CCDC85B
KRT17	pp	CCDC85B
KRT6A	pp	CCDC85B
MCM10	pp	CCDC85B
PKN1	pp	CCDC85B
SMARCD1	pp	CCDC85B
CCNA1	pp	CCNA1
CDC6	pp	CCNA2
TAF1	pp	CCNA2
TP53	pp	CCNA2
FLNA	pp	CCNB1
GADD45B	pp	CCNB1
GADD45G	pp	CCNB1
KAT5	pp	CCNB1
MAP4	pp	CCNB1
PCNA	pp	CCNB1

RUNX2	pp	CCNB1
TGFBR2	pp	CCNB1
TSC1	pp	CCNB1
AR	pp	CCND1
CDK6	pp	CCND1
GSK3B	pp	CCND1
KAT2B	pp	CCND1
MCM10	pp	CCND1
NCOA1	pp	CCND1
PCNA	pp	CCND1
PRKACA	pp	CCND1
RB1	pp	CCND1
RUNX1	pp	CCND1
SP1	pp	CCND1
TAF1	pp	CCND1
THRA	pp	CCND1
THRB	pp	CCND1
CDKN1A	pp	CCND2
RUNX1	pp	CCND2
ATP5C1	pp	CCND3
CDK14	pp	CCND3
CDK6	pp	CCND3
MCM10	pp	CCND3
PCNA	pp	CCND3
RUNX1	pp	CCND3
AR	pp	CCNE1
CDK2	pp	CCNE1
COIL	pp	CCNE1
FBXW7	pp	CCNE1
GSK3B	pp	CCNE1
SMARCD3	pp	CCNE1
CDKN1C	pp	CCNE2
SUMO2	pp	CCNE2
SUMO3	pp	CCNE2
CD44	pp	CD44
COL14A1	pp	CD44
COL1A1	pp	CD44
COL1A2	pp	CD44
EGFR	pp	CD44
ERBB4	pp	CD44
EZR	pp	CD44
FGF2	pp	CD44
IGFBP3	pp	CD44
LCK	pp	CD44

MMP1	pp	CD44
MMP7	pp	CD44
NF2	pp	CD44
SPP1	pp	CD44
TGFBR1	pp	CD44
TGFBR2	pp	CD44
BNIP3	pp	CD47
UBQLN1	pp	CD47
ADAM2	pp	CD9
KIT	pp	CD9
PRKCA	pp	CD9
ARHGDIB	pp	CDC42
CASP3	pp	CDC42
CSPG4	pp	CDC42
FLNA	pp	CDC42
KAT5	pp	CDC42
MAP3K4	pp	CDC42
PAK4	pp	CDC42
PIK3R1	pp	CDC42
PRKCA	pp	CDC42
PRKCG	pp	CDC42
PRKCZ	pp	CDC42
RPS6KB1	pp	CDC42
TBC1D3F	pp	CDC42
TNK2	pp	CDC42
MCM10	pp	CDC45L
YWHAG	pp	CDC5L
CDK2	pp	CDC6
MCM10	pp	CDC6
CDK2	pp	CDC7
MCM10	pp	CDC7
CA9	pp	CDH1
CASP3	pp	CDH1
CDH1	pp	CDH1
CSNK2A1	pp	CDH1
CTNNA1	pp	CDH1
GSK3B	pp	CDH1
HDAC1	pp	CDH1
HDAC2	pp	CDH1
NDRG1	pp	CDH1
PPP1CA	pp	CDH1
PSEN1	pp	CDH1
EPHA2	pp	CDH5
BIRC6	pp	CDK1

CCNA1	pp	CDK1
CCNA2	pp	CDK1
CCNB1	pp	CDK1
CCNB2	pp	CDK1
CCNE1	pp	CDK1
CDK1	pp	CDK1
GADD45A	pp	CDK1
GADD45B	pp	CDK1
GADD45G	pp	CDK1
KAT5	pp	CDK1
LYN	pp	CDK1
MYC	pp	CDK1
PCNA	pp	CDK1
PPP2R1A	pp	CDK1
PPP2R1B	pp	CDK1
TLE1	pp	CDK1
TP53	pp	CDK1
ACVR1	pp	CDK14
BMPR1B	pp	CDK14
RB1	pp	CDK14
BCL2	pp	CDK2
CCNA1	pp	CDK2
CCNA2	pp	CDK2
CCNB2	pp	CDK2
CCNE2	pp	CDK2
CDK2	pp	CDK2
HIST1H1B	pp	CDK2
LYN	pp	CDK2
PCNA	pp	CDK2
CCNA2	pp	CDK3
CCNE1	pp	CDK3
CCNE2	pp	CDK3
CDK3	pp	CDK3
BIRC5	pp	CDK4
BMPR1B	pp	CDK4
CCND1	pp	CDK4
CCND2	pp	CDK4
CCND3	pp	CDK4
CDC45L	pp	CDK4
CDC7	pp	CDK4
CDKN1C	pp	CDK4
PPP2R1B	pp	CDK4
ABL1	pp	CDK5
CCND2	pp	CDK5

CDK5	pp	CDK5
CSNK1D	pp	CDK5
GSK3B	pp	CDK5
PCNA	pp	CDK5
SET	pp	CDK5
SYNJ1	pp	CDK5
AR	pp	CDK6
CDK6	pp	CDK6
CTNNB1	pp	CDK6
MCM10	pp	CDK6
RB1	pp	CDK6
RUNX1	pp	CDK6
AKT1	pp	CDKN1A
BCCIP	pp	CDKN1A
CASP3	pp	CDKN1A
CCNA1	pp	CDKN1A
CCNA2	pp	CDKN1A
CCNB1	pp	CDKN1A
CCNB2	pp	CDKN1A
CCND1	pp	CDKN1A
CCND3	pp	CDKN1A
CCNE1	pp	CDKN1A
CCNE2	pp	CDKN1A
CDC45L	pp	CDKN1A
CDC5L	pp	CDKN1A
CDC6	pp	CDKN1A
CDC7	pp	CDKN1A
CDK1	pp	CDKN1A
CDK14	pp	CDKN1A
CDK2	pp	CDKN1A
CDK3	pp	CDKN1A
CDK4	pp	CDKN1A
CDK6	pp	CDKN1A
CDKN1A	pp	CDKN1A
CIZ1	pp	CDKN1A
DAPK3	pp	CDKN1A
ESR1	pp	CDKN1A
GADD45A	pp	CDKN1A
GADD45B	pp	CDKN1A
GADD45G	pp	CDKN1A
MAPK8	pp	CDKN1A
MCM10	pp	CDKN1A
PARP1	pp	CDKN1A
PCNA	pp	CDKN1A

PIM1	pp	CDKN1A
POLD2	pp	CDKN1A
PSMA3	pp	CDKN1A
RB1	pp	CDKN1A
RNF144B	pp	CDKN1A
SET	pp	CDKN1A
TK1	pp	CDKN1A
TSG101	pp	CDKN1A
XRCC6	pp	CDKN1A
LIMK1	pp	CDKN1C
NR4A2	pp	CDKN1C
ATF2	pp	CEBPA
CDK2	pp	CEBPA
CDK4	pp	CEBPA
CDKN1A	pp	CEBPA
CEBPA	pp	CEBPA
GSK3B	pp	CEBPA
HNF1A	pp	CEBPA
MYC	pp	CEBPA
NR3C1	pp	CEBPA
RB1	pp	CEBPA
RUNX1	pp	CEBPA
SOX10	pp	CEBPA
SOX8	pp	CEBPA
SUMO3	pp	CEBPA
CEBPG	pp	CEBPG
DDIT3	pp	CEBPG
FOS	pp	CEBPG
GTF2F2	pp	CEBPG
CFTR	pp	CFTR
PPP2R1B	pp	CFTR
PRKACA	pp	CFTR
PRKCA	pp	CFTR
SLC9A3R1	pp	CFTR
SLC9A3R2	pp	CFTR
BMP2	pp	CHRD
AKT1	pp	CHUK
BCL10	pp	CHUK
CHUK	pp	CHUK
CREBBP	pp	CHUK
IKBKG	pp	CHUK
MAP3K7	pp	CHUK
NFKB1	pp	CHUK
NFKBIA	pp	CHUK

NOTCH3	pp	CHUK
PRKCB	pp	CHUK
PRKDC	pp	CHUK
PTPN11	pp	CHUK
TRAF2	pp	CHUK
AP1G1	pp	CLTC
CLTC	pp	CLTC
HIP1	pp	CLTC
KIT	pp	CLTC
NDRG1	pp	CLTC
TNK2	pp	CLTC
XRCC6	pp	CLTC
YWHAG	pp	CLTC
CNDP2	pp	CNDP2
NDRG1	pp	CNDP2
CNOT1	pp	CNOT2
CNOT1	pp	CNOT3
CNOT2	pp	CNOT3
CNOT8	pp	CNOT3
CREBBP	pp	CNOT3
CNOT1	pp	CNOT8
CNOT2	pp	CNOT8
CNTN1	pp	CNTN1
ATXN1	pp	COIL
C1QBP	pp	COIL
CDK2	pp	COIL
COIL	pp	COIL
CSNK2B	pp	COIL
KPNA3	pp	COIL
PRMT1	pp	COIL
COL13A1	pp	COL13A1
FN1	pp	COL13A1
HSPG2	pp	COL13A1
NID1	pp	COL13A1
NID2	pp	COL13A1
SPARC	pp	COL13A1
DCN	pp	COL14A1
ITGA5	pp	COL18A1
MMP2	pp	COL18A1
DCN	pp	COL1A1
FN1	pp	COL1A1
IGFBP3	pp	COL1A1
ITGA5	pp	COL1A1
MMP2	pp	COL1A1

NID1	pp	COL1A1
P4HB	pp	COL1A1
SPARC	pp	COL1A1
THBS1	pp	COL1A1
DCN	pp	COL1A2
FN1	pp	COL1A2
ITGB3	pp	COL1A2
P4HB	pp	COL1A2
SPARC	pp	COL1A2
C1QA	pp	COL2A1
COL2A1	pp	COL2A1
FN1	pp	COL2A1
MMP1	pp	COL2A1
SPARC	pp	COL2A1
TGFB1	pp	COL2A1
THBS1	pp	COL2A1
COL4A1	pp	COL4A1
DCN	pp	COL4A1
FN1	pp	COL4A1
NID1	pp	COL4A1
DCN	pp	COL4A3
FN1	pp	COL4A3
DCN	pp	COL4A4
FN1	pp	COL4A4
DCN	pp	COL5A1
THBS1	pp	COL5A1
NDRG1	pp	COPB2
COPS4	pp	COPS4
RCBTB2	pp	COPS4
COPS4	pp	COPS5
ESR1	pp	COPS5
HNF4A	pp	COPS5
NR4A2	pp	COPS5
PPARG	pp	COPS5
SMAD2	pp	COPS5
ARAF	pp	CPS1
ACVR1	pp	CREBBP
CEBPA	pp	CREBBP
CREBBP	pp	CREBBP
ELK1	pp	CREBBP
ETS1	pp	CREBBP
ETS2	pp	CREBBP
HIF1A	pp	CREBBP
HNF4A	pp	CREBBP

HSF1	pp	CREBBP
IKBKG	pp	CREBBP
KAT2B	pp	CREBBP
KAT5	pp	CREBBP
MAML1	pp	CREBBP
MYC	pp	CREBBP
NCOA1	pp	CREBBP
NFE2	pp	CREBBP
NFE2L2	pp	CREBBP
PRKCD	pp	CREBBP
RPS6KA1	pp	CREBBP
SREBF1	pp	CREBBP
SREBF2	pp	CREBBP
STAT1	pp	CREBBP
THRA	pp	CREBBP
TP73	pp	CREBBP
YWHAB	pp	CRTC2
CSF2	pp	CSF2
KITLG	pp	CSF2
SDC2	pp	CSF2
AKAP9	pp	CSNK1D
ARFGAP1	pp	CSNK1D
DVL1	pp	CSNK1D
PER1	pp	CSNK1D
PER3	pp	CSNK1D
PPP1R14A	pp	CSNK1D
ATF2	pp	CSNK2A1
CDK1	pp	CSNK2A1
CDKN1A	pp	CSNK2A1
CREBBP	pp	CSNK2A1
CSNK2A1	pp	CSNK2A1
CSNK2B	pp	CSNK2A1
DVL1	pp	CSNK2A1
DVL3	pp	CSNK2A1
EEF1D	pp	CSNK2A1
FGF2	pp	CSNK2A1
FOS	pp	CSNK2A1
NCL	pp	CSNK2A1
YWHAB	pp	CSNK2A1
ATF2	pp	CSNK2A2
CREBBP	pp	CSNK2A2
FGF2	pp	CSNK2A2
FOS	pp	CSNK2A2
HSP90AA1	pp	CSNK2A2

MAPK14	pp	CSNK2A2
NCL	pp	CSNK2A2
ARAF	pp	CSNK2B
CDK1	pp	CSNK2B
CDKN1A	pp	CSNK2B
CSNK2A2	pp	CSNK2B
CSNK2B	pp	CSNK2B
LYN	pp	CSNK2B
MAPK14	pp	CSNK2B
PRKCZ	pp	CSNK2B
RPS6KB1	pp	CSNK2B
SHMT2	pp	CSNK2B
BCAR1	pp	CSPG4
MPDZ	pp	CSPG4
TNK2	pp	CSPG4
ERBB4	pp	CTGF
HSPG2	pp	CTGF
NOV	pp	CTGF
TGFB1	pp	CTGF
TGFB2	pp	CTGF
TGFB3	pp	CTGF
VEGFA	pp	CTGF
CTH	pp	CTH
CA9	pp	CTNNA1
CDH5	pp	CTNNA1
CTNNB1	pp	CTNNA1
AR	pp	CTNNB1
BCL3	pp	CTNNB1
CA9	pp	CTNNB1
CASP3	pp	CTNNB1
CCND1	pp	CTNNB1
CDH1	pp	CTNNB1
CDH5	pp	CTNNB1
CEBPA	pp	CTNNB1
CHUK	pp	CTNNB1
CREBBP	pp	CTNNB1
CSNK1D	pp	CTNNB1
CSNK2A1	pp	CTNNB1
CSNK2B	pp	CTNNB1
DVL1	pp	CTNNB1
DVL3	pp	CTNNB1
EGFR	pp	CTNNB1
ERBB2	pp	CTNNB1
ESR1	pp	CTNNB1

EZR	pp	CTNNB1
GSK3B	pp	CTNNB1
HNF1A	pp	CTNNB1
IKBKB	pp	CTNNB1
MITF	pp	CTNNB1
NDRG1	pp	CTNNB1
NF2	pp	CTNNB1
PECAM1	pp	CTNNB1
PIK3R1	pp	CTNNB1
SLC9A3R1	pp	CTNNB1
SMAD2	pp	CTNNB1
SMAD3	pp	CTNNB1
SMAD4	pp	CTNNB1
SPN	pp	CTNNB1
TGFBR1	pp	CTNNB1
FN1	pp	CTSD
PSAP	pp	CTSD
SLC2A4	pp	CTSD
F5	pp	CTSG
LRP1	pp	CTSG
PPBP	pp	CTSG
THBS1	pp	CTSG
COL18A1	pp	CTSL1
CTSL1	pp	CTSL1
RXRA	pp	CTSL1
CA12	pp	CUX1
CDK1	pp	CUX1
CREBBP	pp	CUX1
CTSL1	pp	CUX1
HMGB1	pp	CUX1
KAT2B	pp	CUX1
RB1	pp	CUX1
AGTPBP1	pp	CYCS
APAF1	pp	CYCS
BCL2	pp	CYCS
BCL2L1	pp	CYCS
CASP9	pp	CYCS
RNF7	pp	CYCS
SNCA	pp	CYCS
TRAF6	pp	CYLD
ITGB5	pp	CYR61
DAPK3	pp	DAPK3
MET	pp	DAPK3
ADORA2B	pp	DCC

CASP3	pp	DCC
CASP9	pp	DCC
MAPK3	pp	DCC
PTK2	pp	DCC
SIAH1	pp	DCC
SIAH2	pp	DCC
AHSG	pp	DCN
COL4A5	pp	DCN
COL4A6	pp	DCN
DPT	pp	DCN
EGFR	pp	DCN
ELN	pp	DCN
FBN1	pp	DCN
FLNA	pp	DCN
FN1	pp	DCN
MMP2	pp	DCN
MMP3	pp	DCN
MMP7	pp	DCN
PLA2G2A	pp	DCN
SFTPD	pp	DCN
TGFB1	pp	DCN
THBS1	pp	DCN
TNF	pp	DCN
WISP1	pp	DCN
DCTN1	pp	DCTN1
GSK3B	pp	DCTN1
ATF3	pp	DDIT3
CSNK2A1	pp	DDIT3
FOS	pp	DDIT3
JUN	pp	DDIT3
JUND	pp	DDIT3
MAPK14	pp	DDIT3
NDRG1	pp	DDX1
DDX24	pp	DDX24
EIF3E	pp	DDX24
FADD	pp	DDX24
HSPA5	pp	DDX24
VDAC3	pp	DDX24
CREBBP	pp	DDX5
NDRG1	pp	DDX5
PRKCA	pp	DDX5
SMAD3	pp	DDX5
CYLD	pp	DDX58
MAVS	pp	DDX58

BIRC6	pp	DIABLO
BIRC7	pp	DIABLO
CASP9	pp	DIABLO
DIABLO	pp	DIABLO
AKAP5	pp	DLG1
CTNNA1	pp	DLG1
DLG1	pp	DLG1
DLGAP1	pp	DLG1
MAPK12	pp	DLG1
DLG1	pp	DLG3
DLG4	pp	DLG3
ERBB4	pp	DLG3
AKAP5	pp	DLG4
DLG4	pp	DLG4
DLGAP1	pp	DLG4
ADAM10	pp	DLL1
DLL1	pp	DLL1
NDRG1	pp	DLST
AP1G1	pp	DNER
BNIP3L	pp	DOK5
CCDC85B	pp	DOK5
LOX	pp	DPT
NFIL3	pp	DR1
CASP10	pp	DSP
CASP9	pp	DSP
CDH5	pp	DSP
DSP	pp	DSP
PECAM1	pp	DSP
PRKACA	pp	DSP
VIM	pp	DSP
MAPK3	pp	DUSP3
STAT1	pp	DUSP3
DVL1	pp	DVL1
SMAD2	pp	DVL1
CSNK1D	pp	DVL3
DVL1	pp	DVL3
DVL3	pp	DVL3
CDK1	pp	EEF1D
EEF1D	pp	EEF1D
EEF1G	pp	EEF1D
PRKCA	pp	EEF1D
RPS6KA1	pp	EEF1D
CDC42	pp	EEF1G
EEF1G	pp	EEF1G

NDRG1	pp	EEF1G
SKIL	pp	EEF1G
SNAPIN	pp	EEF1G
EEF2	pp	EEF2
NDRG1	pp	EEF2
PPP2CA	pp	EEF2
PPP2R1A	pp	EEF2
PPP2R1B	pp	EEF2
TP53	pp	EEF2
ADAM10	pp	EFNA1
EEF1G	pp	EFNA1
EPHA4	pp	EFNA1
EPHA5	pp	EFNA1
EPHA6	pp	EFNA1
EPHA8	pp	EFNA1
KAT5	pp	EFNA1
EPHA4	pp	EFNA3
EPHA7	pp	EFNA3
ADAM12	pp	EGF
ADRBK1	pp	EGF
DUSP3	pp	EGF
EGF	pp	EGF
EGFR	pp	EGF
ERBB2	pp	EGF
ERBB3	pp	EGF
LMNA	pp	EGF
NRG1	pp	EGF
AGTR1	pp	EGFR
ANXA1	pp	EGFR
ATP5C1	pp	EGFR
CDH1	pp	EGFR
EGFR	pp	EGFR
ERBB3	pp	EGFR
ESR1	pp	EGFR
HTT	pp	EGFR
ITGA5	pp	EGFR
KRT17	pp	EGFR
KRT7	pp	EGFR
PIK3R1	pp	EGFR
PRKACA	pp	EGFR
PRKCA	pp	EGFR
PRKCD	pp	EGFR
PTK2	pp	EGFR
PTPN11	pp	EGFR

RIPK1	pp	EGFR
TNC	pp	EGFR
TNK2	pp	EGFR
XRCC6	pp	EGFR
YWHAZ	pp	EGFR
EPAS1	pp	EGLN3
HIF1A	pp	EGLN3
HIF3A	pp	EGLN3
OS9	pp	EGLN3
EIF2B3	pp	EIF2B3
STC2	pp	EIF2B3
NDRG1	pp	EIF2S3
EPAS1	pp	EIF3E
NDRG1	pp	EIF3E
MAP2K1	pp	ELK1
MAPK14	pp	ELK1
MAPK3	pp	ELK1
SRF	pp	ELK1
TAOK2	pp	ELK1
SRF	pp	ELK4
FBN1	pp	ELN
LOX	pp	ELN
NID2	pp	ELN
SPINK1	pp	ELN
ENG	pp	ENG
TGFB1	pp	ENG
TGFB2	pp	ENG
TGFB3	pp	ENG
TGFBR1	pp	ENG
TGFBR2	pp	ENG
TGFBR3	pp	ENG
ANXA11	pp	ENO2
SUMO4	pp	ENO2
ENO2	pp	ENSA
PRKD1	pp	EPAS1
SMAD3	pp	EPAS1
EFNA1	pp	EPHA1
EFNA1	pp	EPHA2
EFNA3	pp	EPHA2
EGFR	pp	EPHA2
EPHA2	pp	EPHA2
PTK2	pp	EPHA2
EFNA1	pp	EPHA3
EPHA3	pp	EPHA3

PIM1	pp	EPHA3
TP53	pp	EPHA3
EPHA4	pp	EPHA4
EFNA3	pp	EPHA5
EFNA1	pp	EPHA7
EPHA7	pp	EPHA7
EPHA8	pp	EPHA8
EFNA1	pp	EPHB1
EPHB1	pp	EPHB1
EPHB2	pp	EPHB2
MMP2	pp	EPHB2
SYNJ1	pp	EPHB2
VEGFA	pp	EPHB2
EPOR	pp	EPOR
GNB2L1	pp	EPOR
KIT	pp	EPOR
KITLG	pp	EPOR
KLK3	pp	EPOR
LYN	pp	EPOR
MAPK1	pp	EPOR
MAPK3	pp	EPOR
PIK3R1	pp	EPOR
PTPN11	pp	EPOR
CAV1	pp	ERBB2
DLG4	pp	ERBB2
EGFR	pp	ERBB2
ERBB2	pp	ERBB2
ERBB3	pp	ERBB2
ERBB4	pp	ERBB2
ESR1	pp	ERBB2
ITGA5	pp	ERBB2
NF2	pp	ERBB2
PIK3R1	pp	ERBB2
PTK2	pp	ERBB2
CDK5	pp	ERBB3
ERBB3	pp	ERBB3
ERBB4	pp	ERBB3
PTK2	pp	ERBB3
DLG1	pp	ERBB4
DLG4	pp	ERBB4
EGFR	pp	ERBB4
ERBB4	pp	ERBB4
ERC2	pp	ERC2
PPFIA4	pp	ERC2

ERO1L	pp	ERO1L
PDIA3	pp	ERO1L
PDILT	pp	ERO1L
ERO1L	pp	ERP44
AKT1	pp	ESR1
AR	pp	ESR1
CAV1	pp	ESR1
CCND1	pp	ESR1
CHUK	pp	ESR1
COBRA1	pp	ESR1
CREBBP	pp	ESR1
CSNK2B	pp	ESR1
DDX5	pp	ESR1
ESR1	pp	ESR1
ESR2	pp	ESR1
GADD45B	pp	ESR1
GADD45G	pp	ESR1
HSP90AA1	pp	ESR1
JUN	pp	ESR1
LCK	pp	ESR1
MAPK1	pp	ESR1
MAPK14	pp	ESR1
MED1	pp	ESR1
MNAT1	pp	ESR1
NCOA1	pp	ESR1
NCOR2	pp	ESR1
NR1H4	pp	ESR1
PGR	pp	ESR1
PIK3R1	pp	ESR1
PPARGC1A	pp	ESR1
RBM9	pp	ESR1
RPS6KA1	pp	ESR1
SMAD2	pp	ESR1
SMARCD3	pp	ESR1
TBP	pp	ESR1
TP53	pp	ESR1
XBP1	pp	ESR1
AKT1	pp	ESR2
ESR2	pp	ESR2
LCK	pp	ESR2
MED1	pp	ESR2
NCOA1	pp	ESR2
NCOR2	pp	ESR2
RBM9	pp	ESR2

SMAD2	pp	ESR2
SMAD4	pp	ESR2
SREBF1	pp	ESR2
ESR1	pp	ESRRA
ESRRA	pp	ESRRA
ESRRG	pp	ESRRA
EXOSC6	pp	ESRRA
NR0B1	pp	ESRRA
ESRRA	pp	ESRRG
PPARGC1A	pp	ESRRG
ETS1	pp	ETS1
ETS2	pp	ETS1
FOS	pp	ETS1
JUN	pp	ETS1
MAF	pp	ETS1
MAPK1	pp	ETS1
MAPK3	pp	ETS1
RUNX2	pp	ETS1
UBE2I	pp	ETS1
ETS2	pp	ETS2
JUN	pp	ETS2
POU5F1	pp	ETS2
CREBBP	pp	EWSR1
EWSR1	pp	EWSR1
NDRG1	pp	EWSR1
POU5F1	pp	EWSR1
PRKCA	pp	EWSR1
TAF1	pp	EWSR1
YWHAG	pp	EWSR1
EXOSC6	pp	EXOSC6
ADORA2B	pp	EZR
ARF6	pp	EZR
ARHGDIB	pp	EZR
CDH1	pp	EZR
CDK5	pp	EZR
CFTR	pp	EZR
CLIC5	pp	EZR
DLG1	pp	EZR
EGFR	pp	EZR
EZR	pp	EZR
FAS	pp	EZR
FASLG	pp	EZR
ICAM1	pp	EZR
ICAM2	pp	EZR

ICAM3	pp	EZR
L1CAM	pp	EZR
LCK	pp	EZR
MSN	pp	EZR
NF2	pp	EZR
PALLD	pp	EZR
PIK3R1	pp	EZR
PRKAR2A	pp	EZR
PRKCA	pp	EZR
PTK2	pp	EZR
PTPRC	pp	EZR
RDX	pp	EZR
ROCK1	pp	EZR
S100P	pp	EZR
SCYL3	pp	EZR
SDC2	pp	EZR
SELL	pp	EZR
SELP	pp	EZR
SLC9A3R1	pp	EZR
SLC9A3R2	pp	EZR
SPN	pp	EZR
TBC1D10A	pp	EZR
TMEM8B	pp	EZR
TSC1	pp	EZR
VCAM1	pp	EZR
WWOX	pp	EZR
F10	pp	F10
CSNK2A1	pp	F11R
F11R	pp	F11R
PRKCA	pp	F11R
F2	pp	F2
F5	pp	F2
F8	pp	F2
IGFBP3	pp	F2
IGFBP5	pp	F2
THBS1	pp	F2
CTSG	pp	F2R
F2	pp	F2R
ITGAV	pp	F2R
PLG	pp	F2R
CSNK2A1	pp	F5
F10	pp	F5
F5	pp	F5
PROC	pp	F5

F10	pp	F8
ANK3	pp	FADD
IKBKG	pp	FADD
RIPK1	pp	FADD
STAT1	pp	FADD
TRAF2	pp	FADD
ANK3	pp	FAS
CD47	pp	FAS
EGFR	pp	FAS
FADD	pp	FAS
FAS	pp	FAS
FASLG	pp	FAS
LCK	pp	FAS
RIPK1	pp	FAS
SUMO1	pp	FAS
TNFSF13	pp	FAS
UBE2I	pp	FAS
FADD	pp	FASLG
FN1	pp	FASLG
LCK	pp	FASLG
MMP7	pp	FASLG
PIK3R1	pp	FASLG
SUMO1	pp	FASLG
EWSR1	pp	FASN
FASN	pp	FASN
NDRG1	pp	FASN
LTBP1	pp	FBN1
PPARGC1A	pp	FBXW7
CSNK2B	pp	FGF2
FGF2	pp	FGF2
HSPG2	pp	FGF2
PF4	pp	FGF2
PRMT1	pp	FGF2
EPHA4	pp	FGFR2
ITGA5	pp	FGFR2
ATF3	pp	FGFR3
C6orf47	pp	FGFR3
EPHA4	pp	FGFR3
FGF2	pp	FGFR3
FGFR3	pp	FGFR3
FKBP1A	pp	FKBP1A
TGFBR1	pp	FKBP1A
YY1	pp	FKBP1A
MAPK14	pp	FKBP8

AR	pp	FLNA
CAV1	pp	FLNA
ERBB3	pp	FLNA
ITGB5	pp	FLNA
LMNA	pp	FLNA
MAP2K4	pp	FLNA
MAPK14	pp	FLNA
MTNR1B	pp	FLNA
PCBP2	pp	FLNA
PSEN2	pp	FLNA
RAC1	pp	FLNA
SMAD3	pp	FLNA
TNIP2	pp	FLNA
TRAF2	pp	FLNA
YWHAG	pp	FLNA
FLT3LG	pp	FLT3LG
FLT4	pp	FLT4
COL4A5	pp	FN1
COL4A6	pp	FN1
IGFBP3	pp	FN1
ITGA5	pp	FN1
ITGB1	pp	FN1
ITGB3	pp	FN1
ITGB6	pp	FN1
KLK3	pp	FN1
LTBP1	pp	FN1
NOV	pp	FN1
REG3A	pp	FN1
SDC2	pp	FN1
SPARC	pp	FN1
TGM2	pp	FN1
THBS1	pp	FN1
TNC	pp	FN1
VHL	pp	FN1
CDC42	pp	FNBP1
FASLG	pp	FNBP1
FNBP1	pp	FNBP1
ATF7	pp	FOS
BATF	pp	FOS
COBRA1	pp	FOS
EEF1D	pp	FOS
ELK1	pp	FOS
ELK4	pp	FOS
EPHB2	pp	FOS

FOS	pp	FOS
GATA4	pp	FOS
GTF2F2	pp	FOS
JUN	pp	FOS
JUND	pp	FOS
MAF	pp	FOS
MAP3K7	pp	FOS
MAPK1	pp	FOS
NCOA1	pp	FOS
NCOR2	pp	FOS
NFATC3	pp	FOS
OPTN	pp	FOS
PRKACA	pp	FOS
PSMC5	pp	FOS
RB1	pp	FOS
RPS6KA1	pp	FOS
RPS6KA4	pp	FOS
RUNX2	pp	FOS
SCHIP1	pp	FOS
SMARCD1	pp	FOS
SUMO1	pp	FOS
SUMO2	pp	FOS
SUMO3	pp	FOS
SUMO4	pp	FOS
TAF1	pp	FOS
TBP	pp	FOS
TRO	pp	FOS
TSC22D3	pp	FOS
USF2	pp	FOS
WDR65	pp	FOS
XBP1	pp	FOS
AP2M1	pp	FURIN
FLNA	pp	FURIN
FURIN	pp	FURIN
EGFR	pp	GAB1
EPOR	pp	GAB1
MAPK1	pp	GAB1
MAPK3	pp	GAB1
MET	pp	GAB1
PTPN11	pp	GAB1
PRKCD	pp	GABRA1
PRKCG	pp	GABRA1
PRKCB	pp	GABRB2
GABRG2	pp	GABRG2

PRKCA pp GABRG2
PRKCB pp GABRG2
GADD45A pp GADD45A
HIST2H2BE pp GADD45A
PCNA pp GADD45A
PPARA pp GADD45A
RXRA pp GADD45A
GADD45G pp GADD45B
MAP2K7 pp GADD45B
C1orf103 pp GADD45G
EEF1G pp GADD45G
KAT5 pp GADD45G
PIAS4 pp GADD45G
AR pp GAPDH
ATXN1 pp GAPDH
EGFR pp GAPDH
GAPDH pp GAPDH
HTT pp GAPDH
KAT5 pp GAPDH
NR1H4 pp GAPDH
PGK1 pp GAPDH
PLD2 pp GAPDH
SLC2A4 pp GAPDH
SUMO4 pp GAPDH
GDF15 pp GDF15
GIPC1 pp GIPC1
C1orf103 pp GIT1
DDX24 pp GIT1
GIT1 pp GIT1
PPFIA4 pp GIT1
TERF1 pp GIT1
YWHAG pp GIT1
CAV1 pp GJA1
CSNK1D pp GJA1
MAPK1 pp GJA1
MAPK3 pp GJA1
NOV pp GJA1
PRKCA pp GJA1
PRKCB pp GJA1
PRKCE pp GJA1
PRKCG pp GJA1
CREBBP pp GMEB1
GMEB1 pp GMEB1
SMAD3 pp GMEB1

CDKN1A	pp	GMNN
AR	pp	GNB2L1
CCNA1	pp	GNB2L1
CDKN1A	pp	GNB2L1
GNB2L1	pp	GNB2L1
HABP4	pp	GNB2L1
PABPC1	pp	GNB2L1
PDE4D	pp	GNB2L1
PTN	pp	GNB2L1
RB1	pp	GNB2L1
STAT1	pp	GNB2L1
SUMO4	pp	GNB2L1
TP73	pp	GNB2L1
TUBB	pp	GNB2L1
CSNK2A1	pp	GPI
GPI	pp	GPI
BMP2	pp	GREM1
YWHAH	pp	GREM1
DLG3	pp	GRIN2C
DLG4	pp	GRIN2C
DLGAP1	pp	GRIN2C
IL16	pp	GRIN2C
INADL	pp	GRIN2C
AR	pp	GRIP1
CSPG4	pp	GRIP1
CSPG4	pp	GRIP2
AGTR1	pp	GRK5
CAV1	pp	GRK5
GIT1	pp	GRK5
GRK5	pp	GRK5
GIT1	pp	GRK6
GRK6	pp	GRK6
AKT1	pp	GSK3B
AR	pp	GSK3B
BCL3	pp	GSK3B
GSK3B	pp	GSK3B
IKBKG	pp	GSK3B
MYC	pp	GSK3B
PPARGC1A	pp	GSK3B
PRKACA	pp	GSK3B
PRKCZ	pp	GSK3B
GSTP1	pp	GSTP1
MAPK8	pp	GSTP1
SUMO4	pp	GSTP1

TGM2	pp	GSTP1
AR	pp	GTF2F2
GTF2F2	pp	GTF2F2
TBP	pp	GTF2F2
FGFR3	pp	GTF3C1
GZMB	pp	GZMB
ENO2	pp	HABP4
PRKCA	pp	HABP4
PRKCB	pp	HABP4
PRKCD	pp	HABP4
PRKCG	pp	HABP4
PRKCZ	pp	HABP4
PRMT1	pp	HABP4
VIM	pp	HABP4
CREB3	pp	HCFC1
HCFC1	pp	HCFC1
PPARGC1A	pp	HCFC1
ZBTB17	pp	HCFC1
CSNK2A1	pp	HCLS1
LYN	pp	HCLS1
BCL3	pp	HDAC1
CBFA2T3	pp	HDAC1
CREBBP	pp	HDAC1
CSNK2A1	pp	HDAC1
CSNK2A2	pp	HDAC1
HDAC1	pp	HDAC1
HDAC2	pp	HDAC1
HIF1A	pp	HDAC1
KAT5	pp	HDAC1
KDM1A	pp	HDAC1
PHF21A	pp	HDAC1
PIAS4	pp	HDAC1
PPP2R1B	pp	HDAC1
RBPJ	pp	HDAC1
RUNX1T1	pp	HDAC1
SIN3A	pp	HDAC1
SMAD2	pp	HDAC1
TP53	pp	HDAC1
UBE2I	pp	HDAC1
CDKN1A	pp	HDAC11
PHYHIP	pp	HDAC11
CSNK2A1	pp	HDAC2
CSNK2A2	pp	HDAC2
IKZF4	pp	HDAC2

PHF21A	pp	HDAC2
RCOR1	pp	HDAC2
SIN3A	pp	HDAC2
THRA	pp	HDAC2
THRB	pp	HDAC2
TP53	pp	HDAC2
FN1	pp	HGF
HGF	pp	HGF
KLKB1	pp	HGF
MET	pp	HGF
SDC2	pp	HGF
COPS5	pp	HIF1A
CSNK2A1	pp	HIF1A
CTNNB1	pp	HIF1A
HIF3A	pp	HIF1A
OS9	pp	HIF1A
STAT3	pp	HIF1A
HIP1	pp	HIP1
LOX	pp	HIST1H1B
SIRT1	pp	HIST1H1B
TFAP2B	pp	HIST1H2AC
AKT1	pp	HIST2H2BE
ATF2	pp	HIST2H2BE
ENO2	pp	HK1
SLC2A4	pp	HK2
ERBB2	pp	HLA-A
HLA-A	pp	HLA-A
KIR3DL2	pp	HLA-A
PDIA3	pp	HLA-A
PRKCA	pp	HLA-A
PHF21A	pp	HMG20B
VIM	pp	HMG20B
AES	pp	HMGB1
AR	pp	HMGB1
CASP3	pp	HMGB1
HNRNPK	pp	HMGB1
NFKB1	pp	HMGB1
PLG	pp	HMGB1
POU5F1	pp	HMGB1
PRKCA	pp	HMGB1
PRKDC	pp	HMGB1
RB1	pp	HMGB1
RELA	pp	HMGB1
TAF1	pp	HMGB1

TBP pp HMGB1
TLE1 pp HMGB1
TLE2 pp HMGB1
TP53 pp HMGB1
TP73 pp HMGB1
PGR pp HMGB2
POU5F1 pp HMGB2
PRKDC pp HMGB2
SET pp HMGB2
TP53 pp HMGB2
CREBBP pp HNF1A
FOS pp HNF1A
HNF1A pp HNF1A
HNF4A pp HNF1A
KAT2B pp HNF1A
TLE1 pp HNF1A
ESR1 pp HNF4A
HNF4A pp HNF4A
MAPK14 pp HNF4A
MED1 pp HNF4A
SMAD2 pp HNF4A
SMAD3 pp HNF4A
SMAD4 pp HNF4A
SREBF2 pp HNF4A
NDRG1 pp HNRNPF
SUMO4 pp HNRNPF
HNRNPF pp HNRNPH1
NDRG1 pp HNRNPH1
SUMO4 pp HNRNPH1
YWHAG pp HNRNPH1
DDX1 pp HNRNPK
HNRNPK pp HNRNPK
PCBP2 pp HNRNPK
PRKCD pp HNRNPK
PRMT1 pp HNRNPK
ACTB pp HNRNPU
CASP3 pp HNRNPU
NDRG1 pp HNRNPU
PRMT1 pp HNRNPU
PTPN11 pp HNRNPU
ANK3 pp HOOK1
HOOK1 pp HOOK1
MAFF pp HOXD12
NDRG1 pp HSD17B4

CSNK2A1	pp	HSF1
ENO2	pp	HSF1
HSF1	pp	HSF1
MAPK3	pp	HSF1
MAPK8	pp	HSF1
PRKDC	pp	HSF1
STAT1	pp	HSF1
SUMO1	pp	HSF1
TAF1	pp	HSF1
TBP	pp	HSF1
XRCC5	pp	HSF1
XRCC6	pp	HSF1
AKT1	pp	HSP90AA1
AR	pp	HSP90AA1
CHUK	pp	HSP90AA1
CSNK2A1	pp	HSP90AA1
ERBB2	pp	HSP90AA1
HIF1A	pp	HSP90AA1
HSP90AA1	pp	HSP90AA1
IKBKB	pp	HSP90AA1
IKBKG	pp	HSP90AA1
MAPK1	pp	HSP90AA1
MYC	pp	HSP90AA1
NDRG1	pp	HSP90AA1
NOS3	pp	HSP90AA1
PIM1	pp	HSP90AA1
PPARA	pp	HSP90AA1
PRKDC	pp	HSP90AA1
RAF1	pp	HSP90AA1
RIPK1	pp	HSP90AA1
SLC2A4	pp	HSP90AA1
TP53	pp	HSP90AA1
F8	pp	HSPA5
MTNR1B	pp	HSPA5
NDRG1	pp	HSPA5
SUMO4	pp	HSPA5
YWHAB	pp	HSPA5
CASP3	pp	HSPD1
CASP9	pp	HSPD1
HIST2H2BE	pp	HSPD1
HSPD1	pp	HSPD1
ITGB1	pp	HSPD1
PPP2R1A	pp	HSPD1
PPP2R1B	pp	HSPD1

PRKACA	pp	HSPD1
SUMO4	pp	HSPD1
YWHAG	pp	HSPD1
FBN1	pp	HSPG2
ITGB1	pp	HSPG2
NID2	pp	HSPG2
PDGFA	pp	HSPG2
SPARC	pp	HSPG2
CSNK2A1	pp	HSPH1
CSNK2A2	pp	HSPH1
AKT1	pp	HTT
CASP3	pp	HTT
CREBBP	pp	HTT
DLG4	pp	HTT
GIT1	pp	HTT
HIP1	pp	HTT
OPTN	pp	HTT
PIAS4	pp	HTT
SIN3A	pp	HTT
SP1	pp	HTT
TBP	pp	HTT
TGM2	pp	HTT
TP53	pp	HTT
TUBB	pp	HTT
XRCC6	pp	HTT
EGFR	pp	ICAM1
ICAM1	pp	ICAM1
IL2RG	pp	ICAM1
MSN	pp	ICAM1
SPN	pp	ICAM1
MSN	pp	ICAM2
RDX	pp	ICAM2
MSN	pp	ICAM3
ITGAV	pp	ICAM4
ITGB1	pp	ICAM4
CDK2	pp	ID3
ELK1	pp	ID3
ELK4	pp	ID3
ID3	pp	ID3
MAPK1	pp	IER3
EIF3E	pp	IFIT1
IFIT1	pp	IFIT3
IFIT2	pp	IFIT3
IFIT3	pp	IFIT3

IGFALS	pp	IGF1
NOV	pp	IGF1
DOK5	pp	IGF1R
ESR1	pp	IGF1R
GIPC1	pp	IGF1R
GNB2L1	pp	IGF1R
IGF1	pp	IGF1R
IGF1R	pp	IGF1R
IGF2	pp	IGF1R
ITGB1	pp	IGF1R
PIK3R1	pp	IGF1R
PTPN11	pp	IGF1R
STAT3	pp	IGF1R
YWHAB	pp	IGF1R
YWHAE	pp	IGF1R
YWHAG	pp	IGF1R
YWHAZ	pp	IGF1R
NOV	pp	IGF2
IGF1	pp	IGFBP1
IGF2	pp	IGFBP1
MMP26	pp	IGFBP1
TF	pp	IGFBP1
ADAM12	pp	IGFBP3
CSNK2A1	pp	IGFBP3
CTSD	pp	IGFBP3
CTSG	pp	IGFBP3
CTSL1	pp	IGFBP3
GPI	pp	IGFBP3
GTF3C1	pp	IGFBP3
IGF1	pp	IGFBP3
IGF1R	pp	IGFBP3
IGF2	pp	IGFBP3
IGFALS	pp	IGFBP3
KLK3	pp	IGFBP3
KLKB1	pp	IGFBP3
KPNB1	pp	IGFBP3
LTBP1	pp	IGFBP3
MMP1	pp	IGFBP3
MMP2	pp	IGFBP3
MMP3	pp	IGFBP3
PAPPA2	pp	IGFBP3
RXRA	pp	IGFBP3
TF	pp	IGFBP3
ADAM12	pp	IGFBP5

FN1	pp	IGFBP5
IGF1	pp	IGFBP5
IGF2	pp	IGFBP5
IGFALS	pp	IGFBP5
KPNB1	pp	IGFBP5
SPP1	pp	IGFBP5
TF	pp	IGFBP5
THBS1	pp	IGFBP5
ATF3	pp	IGSF21
IGSF21	pp	IGSF21
PRMT1	pp	IGSF21
TMSB4X	pp	IGSF21
ACVR1	pp	IKBKB
AKT1	pp	IKBKB
CHUK	pp	IKBKB
IKBKB	pp	IKBKB
IKBKG	pp	IKBKB
MAP3K7	pp	IKBKB
PRKCB	pp	IKBKB
PRKCZ	pp	IKBKB
BCL10	pp	IKBKG
CARD8	pp	IKBKG
CYLD	pp	IKBKG
IKBKG	pp	IKBKG
NFKB1	pp	IKBKG
NFKBIA	pp	IKBKG
RIPK1	pp	IKBKG
RNF11	pp	IKBKG
TNIP2	pp	IKBKG
ZFAND5	pp	IKBKG
IKZF4	pp	IKZF4
NRG1	pp	IKZF4
SIN3A	pp	IKZF4
SIN3B	pp	IKZF4
IL15RA	pp	IL15
TRAF2	pp	IL15RA
CASP3	pp	IL16
CDK1	pp	IL16
CSNK2A1	pp	IL16
CSNK2A2	pp	IL16
IL16	pp	IL16
ENO2	pp	IL17B
FNDC6	pp	IL20
GNB2L1	pp	IL2RB

IL15	pp	IL2RB
IL15RA	pp	IL2RB
IL2RG	pp	IL2RB
LCK	pp	IL2RB
PIK3R1	pp	IL2RB
STAT1	pp	IL2RB
IL15	pp	IL2RG
IL15RA	pp	IL2RG
STAT1	pp	IL2RG
NDRG1	pp	ILF3
CAV1	pp	ILK
GSK3B	pp	ILK
MAPK12	pp	INADL
PRDM1	pp	IRF4
TRAF2	pp	IRF4
ANGPT1	pp	ITGA5
ANGPTL3	pp	ITGA5
ARHGAP5	pp	ITGA5
AUP1	pp	ITGA5
CD9	pp	ITGA5
FLT4	pp	ITGA5
GIPC1	pp	ITGA5
ITGB1	pp	ITGA5
ITGB3	pp	ITGA5
ITGB5	pp	ITGA5
L1CAM	pp	ITGA5
NISCH	pp	ITGA5
PECAM1	pp	ITGA5
SPP1	pp	ITGA5
TNC	pp	ITGA5
ADAM9	pp	ITGAV
ANGPTL3	pp	ITGAV
AUP1	pp	ITGAV
AZGP1	pp	ITGAV
CD47	pp	ITGAV
CYR61	pp	ITGAV
EDIL3	pp	ITGAV
ITGB8	pp	ITGAV
L1CAM	pp	ITGAV
MMP14	pp	ITGAV
P2RY2	pp	ITGAV
PAK4	pp	ITGAV
PLP1	pp	ITGAV
PTK2	pp	ITGAV

THY1	pp	ITGAV
ARHGAP5	pp	ITGB1
CD9	pp	ITGB1
FLNA	pp	ITGB1
FLT4	pp	ITGB1
GNB2L1	pp	ITGB1
ILK	pp	ITGB1
ITGAV	pp	ITGB1
ITGB1	pp	ITGB1
ITGB1BP3	pp	ITGB1
NF2	pp	ITGB1
PRKCA	pp	ITGB1
PRKCE	pp	ITGB1
PTK2	pp	ITGB1
SLC3A2	pp	ITGB1
SPP1	pp	ITGB1
TGM2	pp	ITGB1
VCAM1	pp	ITGB1
YWHAB	pp	ITGB1
CDKN1A	pp	ITGB1BP3
AKT1	pp	ITGB3
ANGPTL3	pp	ITGB3
ILK	pp	ITGB3
ITGAV	pp	ITGB3
ITGB3	pp	ITGB3
P2RY2	pp	ITGB3
PDGFRA	pp	ITGB3
PTK2	pp	ITGB3
TGM2	pp	ITGB3
THBS1	pp	ITGB3
THY1	pp	ITGB3
GNB2L1	pp	ITGB5
ITGAV	pp	ITGB5
ITGB5	pp	ITGB5
LTBP1	pp	ITGB5
P2RY2	pp	ITGB5
PAK4	pp	ITGB5
ZBTB17	pp	ITGB5
ITGAV	pp	ITGB6
TGFB1	pp	ITGB6
FURIN	pp	ITM2C
MTNR1B	pp	ITM2C
RNF7	pp	ITM2C
STT3A	pp	ITM2C

ABL1 pp JUN
AR pp JUN
ATF3 pp JUN
BATF pp JUN
COBRA1 pp JUN
COPS5 pp JUN
CREBBP pp JUN
CSNK2A1 pp JUN
FBXW7 pp JUN
FOSL2 pp JUN
GTF2F2 pp JUN
HCFC1 pp JUN
HIF1A pp JUN
JUN pp JUN
MAF pp JUN
MAPK3 pp JUN
MAPK8 pp JUN
MYBBP1A pp JUN
NCOA1 pp JUN
PRKD1 pp JUN
PRKDC pp JUN
RB1 pp JUN
RUNX2 pp JUN
SMAD2 pp JUN
SMAD3 pp JUN
SMAD4 pp JUN
SMARCD1 pp JUN
SMARCD3 pp JUN
SOX10 pp JUN
SOX8 pp JUN
SP1 pp JUN
STAT1 pp JUN
STAT4 pp JUN
SUMO1 pp JUN
SUMO2 pp JUN
SUMO3 pp JUN
SUMO4 pp JUN
TAF1 pp JUN
TBP pp JUN
TSC22D3 pp JUN
UBE2I pp JUN
BATF pp JUNB
ESR1 pp JUNB
FOS pp JUNB

FOSL2	pp	JUNB
JUNB	pp	JUNB
MAPK14	pp	JUNB
MAPK8	pp	JUNB
SMAD3	pp	JUNB
SMAD4	pp	JUNB
ATF3	pp	JUND
BATF	pp	JUND
COPS5	pp	JUND
ESR1	pp	JUND
FOSL2	pp	JUND
MAPK8	pp	JUND
NFE2L2	pp	JUND
SMAD4	pp	JUND
TBP	pp	JUND
AR	pp	KAT2B
KAT2B	pp	KAT2B
SMAD2	pp	KAT2B
SMAD3	pp	KAT2B
TP73	pp	KAT2B
ATXN1	pp	KAT5
ESR1	pp	KAT5
HABP4	pp	KAT5
TP53	pp	KAT5
KCNC1	pp	KCNC1
ALG10	pp	KCNH1
KCNH5	pp	KCNH1
ALG10	pp	KCNH2
PRKACA	pp	KCNH2
YWHAE	pp	KCNH2
KCNQ3	pp	KCNQ3
KCNQ3	pp	KCNQ4
AR	pp	KDM1A
PHF21A	pp	KDM1A
NDRG1	pp	KIF5B
YWHAB	pp	KIF5B
YWHAZ	pp	KIF5B
KIT	pp	KIT
KITLG	pp	KIT
LYN	pp	KIT
MPDZ	pp	KIT
PIK3R1	pp	KIT
PRKCA	pp	KIT
PTPN11	pp	KIT

SPRED1	pp	KIT
CLEC11A	pp	KITLG
CMA1	pp	KITLG
FLT3LG	pp	KITLG
KITLG	pp	KITLG
PIK3CD	pp	KITLG
PGR	pp	KLF9
KPNB1	pp	KPNA3
NFKB1	pp	KPNA3
KPNB1	pp	KPNB1
PIAS4	pp	KPNB1
PLCD1	pp	KPNB1
SMAD2	pp	KPNB1
SMAD3	pp	KPNB1
SNUPN	pp	KPNB1
KRT6A	pp	KRT17
DSP	pp	KRT6A
ATF7IP	pp	KRT7
KRT17	pp	KRT7
LAPTM5	pp	KRT7
CNTN1	pp	L1CAM
CSNK2A1	pp	L1CAM
MSN	pp	L1CAM
RPS6KA1	pp	L1CAM
CEBPZ	pp	LAG3
NID2	pp	LAMC1
BCAR1	pp	LCK
CDC42	pp	LCK
DLG1	pp	LCK
HSP90AA1	pp	LCK
KIT	pp	LCK
LCK	pp	LCK
MAPK1	pp	LCK
MAPK3	pp	LCK
NR3C1	pp	LCK
PECAM1	pp	LCK
PLD2	pp	LCK
PRKACA	pp	LCK
PRKCA	pp	LCK
PTPN11	pp	LCK
PTPRC	pp	LCK
RAF1	pp	LCK
SH2D2A	pp	LCK
THY1	pp	LCK

LDHA	pp	LDHA
NDRG1	pp	LDHA
CA12	pp	LGALS7
LGALS7	pp	LGALS7
LIMK1	pp	LIMK1
PAK4	pp	LIMK1
ROCK1	pp	LIMK1
CDK1	pp	LMNA
FOS	pp	LMNA
KAT5	pp	LMNA
LMNA	pp	LMNA
PRKCA	pp	LMNA
RB1	pp	LMNA
SREBF1	pp	LMNA
SUMO4	pp	LMNA
SMAD9	pp	LNPEP
TNKS	pp	LNPEP
DHDDS	pp	LOX
HIST1H2BJ	pp	LOX
LOX	pp	LOX
BACE1	pp	LRP1
C4BPA	pp	LRP1
CALR	pp	LRP1
CANX	pp	LRP1
CTGF	pp	LRP1
DLG4	pp	LRP1
F8	pp	LRP1
FURIN	pp	LRP1
GIPC1	pp	LRP1
KAT5	pp	LRP1
LRP1	pp	LRP1
MMP14	pp	LRP1
PRKACA	pp	LRP1
THBS1	pp	LRP1
SGOL1	pp	LRRC59
LTBP1	pp	LTBP1
TGM2	pp	LTBP1
BCAR1	pp	LYN
CASP3	pp	LYN
DLG4	pp	LYN
EGFR	pp	LYN
HNRNPK	pp	LYN
IL2RB	pp	LYN
LYN	pp	LYN

MAPK3	pp	LYN
PECAM1	pp	LYN
PRKDC	pp	LYN
PTK2	pp	LYN
PTPRC	pp	LYN
ESR2	pp	MAD2L1
MAD2L1	pp	MAD2L1
UBD	pp	MAD2L1
CREBBP	pp	MAF
MAF	pp	MAF
USF2	pp	MAF
MAFF	pp	MAFF
NFE2	pp	MAFF
PSAP	pp	MAFF
MAML1	pp	MAML1
RBPJ	pp	MAML1
CREBBP	pp	MAML2
NOTCH3	pp	MAML2
NOTCH3	pp	MAML3
MAOA	pp	MAOA
NDRG1	pp	MAOA
ARAF	pp	MAP2K1
BIRC6	pp	MAP2K1
CDK5	pp	MAP2K1
EGFR	pp	MAP2K1
MAP2K1	pp	MAP2K1
MAP3K4	pp	MAP2K1
MAPK1	pp	MAP2K1
MAPK3	pp	MAP2K1
MYC	pp	MAP2K1
PEBP1	pp	MAP2K1
RAF1	pp	MAP2K1
TRAF3	pp	MAP2K1
DUSP3	pp	MAP2K2
MAP2K2	pp	MAP2K2
RAF1	pp	MAP2K2
AKT1	pp	MAP2K4
MAP2K4	pp	MAP2K4
MAP3K2	pp	MAP2K4
MAP3K4	pp	MAP2K4
MAP3K7	pp	MAP2K4
BAD	pp	MAP2K5
DCTN1	pp	MAP2K6
MAP2K6	pp	MAP2K6

MAP3K4	pp	MAP2K6
MAP3K7	pp	MAP2K6
PKN1	pp	MAP2K6
PLCB2	pp	MAP2K6
RELA	pp	MAP2K6
SMAD7	pp	MAP2K6
TAOK2	pp	MAP2K6
MAP2K7	pp	MAP2K7
MAP3K2	pp	MAP2K7
TRAF6	pp	MAP2K7
MAP2K4	pp	MAP3K12
MAP2K7	pp	MAP3K12
MAP2K5	pp	MAP3K2
NFKBIA	pp	MAP3K2
SMURF1	pp	MAP3K2
WNK1	pp	MAP3K2
GADD45A	pp	MAP3K4
GADD45B	pp	MAP3K4
GADD45G	pp	MAP3K4
MAP3K4	pp	MAP3K4
MAPK13	pp	MAP3K4
RAC1	pp	MAP3K4
RUNX2	pp	MAP3K4
AKT1	pp	MAP3K5
CDKN1A	pp	MAP3K5
GADD45B	pp	MAP3K5
IGF1R	pp	MAP3K5
MAP2K6	pp	MAP3K5
MAP2K7	pp	MAP3K5
MAP3K2	pp	MAP3K5
MAP3K5	pp	MAP3K5
MAP3K7	pp	MAP3K5
PPP3R1	pp	MAP3K5
YWHAE	pp	MAP3K5
MAP3K7	pp	MAP3K7
MAPK14	pp	MAP3K7
NAIP	pp	MAP3K7
PEBP1	pp	MAP3K7
RELA	pp	MAP3K7
SMAD7	pp	MAP3K7
CDK1	pp	MAP4
ENO2	pp	MAP4
NR1I3	pp	MAP4
RXRB	pp	MAP4

TUBB	pp	MAP4
C1QBP	pp	MAPK1
DUSP3	pp	MAPK1
EGFR	pp	MAPK1
ELK1	pp	MAPK1
JUND	pp	MAPK1
MAP2K2	pp	MAPK1
MAPK1	pp	MAPK1
MAPK14	pp	MAPK1
MAPK8	pp	MAPK1
MITF	pp	MAPK1
PEBP1	pp	MAPK1
PRKCE	pp	MAPK1
RPS6KA4	pp	MAPK1
RPS6KB1	pp	MAPK1
SREBF2	pp	MAPK1
TNIP1	pp	MAPK1
DLG3	pp	MAPK12
DLG4	pp	MAPK12
MAP2K4	pp	MAPK12
MAP2K6	pp	MAPK12
MAPK12	pp	MAPK12
PKN1	pp	MAPK12
MAP2K6	pp	MAPK13
SMAD4	pp	MAPK13
BCL2	pp	MAPK14
GADD45A	pp	MAPK14
MAP2K1	pp	MAPK14
MAP2K4	pp	MAPK14
MAP2K6	pp	MAPK14
MAPK3	pp	MAPK14
MAPK8	pp	MAPK14
MITF	pp	MAPK14
SMAD7	pp	MAPK14
STAT1	pp	MAPK14
TRAF6	pp	MAPK14
BCL2	pp	MAPK3
BCL3	pp	MAPK3
CAV1	pp	MAPK3
ESR1	pp	MAPK3
GATA4	pp	MAPK3
HIF1A	pp	MAPK3
IER3	pp	MAPK3
JUND	pp	MAPK3

MAP2K2	pp	MAPK3
MAPK3	pp	MAPK3
MAPK8	pp	MAPK3
PTPN11	pp	MAPK3
RPS6KA4	pp	MAPK3
BCL2L1	pp	MAPK8
ELK1	pp	MAPK8
MAP2K4	pp	MAPK8
MAP2K7	pp	MAPK8
MAP3K2	pp	MAPK8
MAP3K7	pp	MAPK8
PRKD1	pp	MAPK8
SMAD3	pp	MAPK8
TP73	pp	MAPK8
TRAF6	pp	MAPK8
WWOX	pp	MAPK8
MAP3K7	pp	MAVS
TICAM1	pp	MAVS
TRAF6	pp	MAVS
CSNK2A1	pp	MAX
EPAS1	pp	MAX
HIF1A	pp	MAX
MAPK14	pp	MAX
MAX	pp	MAX
MXI1	pp	MAX
MYC	pp	MAX
SMAD3	pp	MAX
SMAD4	pp	MAX
ATF7IP	pp	MBD1
OASL	pp	MBD1
CASP3	pp	MCL1
GSK3B	pp	MCL1
MAPK1	pp	MCL1
MCL1	pp	MCL1
CDC5L	pp	MCM10
CCNB2	pp	MCM2
CDC6	pp	MCM2
CDC7	pp	MCM2
CDK4	pp	MCM2
CDK6	pp	MCM2
MCM10	pp	MCM2
MCM2	pp	MCM2
TBP	pp	MCM2
GATA4	pp	MED1

MED1	pp	MED1
NR1I3	pp	MED1
PPARGC1A	pp	MED1
THRA	pp	MED1
THRB	pp	MED1
CTNNB1	pp	MET
EGFR	pp	MET
FAS	pp	MET
MET	pp	MET
PTPN11	pp	MET
PTPRB	pp	MET
FOS	pp	MITF
GSK3B	pp	MITF
SUMO1	pp	MITF
UBE2I	pp	MITF
AFF1	pp	MLLT3
MLLT3	pp	MLLT3
CSNK2B	pp	MME
LYN	pp	MME
NDRG1	pp	MME
PIK3R1	pp	MME
CMA1	pp	MMP1
MMP7	pp	MMP1
BCAR1	pp	MMP14
BACE1	pp	MMP2
HSP90AA1	pp	MMP2
ITGAV	pp	MMP2
MMP14	pp	MMP2
TGFB1	pp	MMP2
THBS1	pp	MMP2
MMP3	pp	MMP3
PLG	pp	MMP3
NT5C2	pp	MOBKL2B
F11R	pp	MPDZ
MPDZ	pp	MPDZ
CA12	pp	MRPL9
MSN	pp	MSN
ROCK1	pp	MSN
SELL	pp	MSN
SLC9A3R1	pp	MSN
SPN	pp	MSN
TSC1	pp	MSN
MTHFD1	pp	MTHFD1
MTNR1B	pp	MTNR1B

SMC3	pp	MXI1
BCL2	pp	MYC
CDC6	pp	MYC
CSNK2A1	pp	MYC
CSNK2A2	pp	MYC
FBXW7	pp	MYC
KAT5	pp	MYC
MAPK3	pp	MYC
MAPK8	pp	MYC
MXI1	pp	MYC
SMAD2	pp	MYC
SMAD3	pp	MYC
TFAP2B	pp	MYC
TP73	pp	MYC
TUBA4A	pp	MYC
YY1	pp	MYC
ZBTB17	pp	MYC
EWSR1	pp	MYOZ2
CASP9	pp	NAIP
NAIP	pp	NAIP
ENO2	pp	NAT9
BNIP3	pp	NCBP1
HNRNPF	pp	NCBP1
HNRNPH1	pp	NCBP1
RPS6KB1	pp	NCBP1
NDRG1	pp	NCL
PARP1	pp	NCL
PPARGC1A	pp	NCL
PRKCZ	pp	NCL
TP53	pp	NCL
COPS5	pp	NCOA1
DDX5	pp	NCOA1
ESRRA	pp	NCOA1
ESRRG	pp	NCOA1
HIF1A	pp	NCOA1
HNF4A	pp	NCOA1
KAT2B	pp	NCOA1
MAPK1	pp	NCOA1
NCOA1	pp	NCOA1
NR1H4	pp	NCOA1
NR1I2	pp	NCOA1
NR1I3	pp	NCOA1
PGR	pp	NCOA1
PPARG	pp	NCOA1

PPARGC1A	pp	NCOA1
PRMT1	pp	NCOA1
SUMO1	pp	NCOA1
AR	pp	NCOR2
CBFA2T2	pp	NCOR2
CHUK	pp	NCOR2
HDAC1	pp	NCOR2
NFKBIA	pp	NCOR2
NR1I2	pp	NCOR2
RUNX1	pp	NCOR2
SKIL	pp	NCOR2
SNW1	pp	NCOR2
SP1	pp	NCOR2
THRA	pp	NCOR2
THRB	pp	NCOR2
ARL4D	pp	NDRG1
PHYHIP	pp	NDRG1
PRKACA	pp	NDRG1
ANXA9	pp	NDUFA4L2
KAT5	pp	NDUFA4L2
ARRB2	pp	NDUFS7
ENO2	pp	NDUFS7
NF2	pp	NF2
SCHIP1	pp	NF2
JUN	pp	NFE2L1
JUNB	pp	NFE2L1
JUND	pp	NFE2L1
MAFF	pp	NFE2L1
CASP3	pp	NFE2L2
JUN	pp	NFE2L2
MAFF	pp	NFE2L2
NFE2	pp	NFE2L2
PPARG	pp	NFE2L2
PRKCD	pp	NFE2L2
CREB3	pp	NFIL3
NFIL3	pp	NFIL3
AR	pp	NFKB1
ATF3	pp	NFKB1
CTNNB1	pp	NFKB1
ESR1	pp	NFKB1
ETS1	pp	NFKB1
FOS	pp	NFKB1
GSK3B	pp	NFKB1
HDAC1	pp	NFKB1

IKBKB	pp	NFKB1
KAT5	pp	NFKB1
NCOA1	pp	NFKB1
NCOR2	pp	NFKB1
NFKB1	pp	NFKB1
PARP1	pp	NFKB1
PRKACA	pp	NFKB1
RIPK1	pp	NFKB1
RPS3	pp	NFKB1
RXRA	pp	NFKB1
SIN3A	pp	NFKB1
TNIP2	pp	NFKB1
TSC22D3	pp	NFKB1
ARRB2	pp	NFKBIA
CAPN2	pp	NFKBIA
CSNK2A1	pp	NFKBIA
HDAC1	pp	NFKBIA
IKBKB	pp	NFKBIA
LCK	pp	NFKBIA
NFKB1	pp	NFKBIA
NFKBIA	pp	NFKBIA
PIK3R1	pp	NFKBIA
RELA	pp	NFKBIA
RPS6KA1	pp	NFKBIA
SUMO1	pp	NFKBIA
SUMO4	pp	NFKBIA
TP53	pp	NFKBIA
VCP	pp	NFKBIA
CEBPZ	pp	NFYB
MYC	pp	NFYB
SP1	pp	NFYB
TBP	pp	NFYB
TP53	pp	NFYB
TP73	pp	NFYB
HSPG2	pp	NID1
ITGAV	pp	NID1
ITGB3	pp	NID1
LAMC1	pp	NID1
NID1	pp	NID1
SKIL	pp	NID1
SKIL	pp	NID2
APAF1	pp	NLRP1
NLRP1	pp	NLRP1
NLRP1	pp	NOD1

NOD1	pp	NOD1
DLG4	pp	NOS1
HSP90AA1	pp	NOS1
PRKACA	pp	NOS1
PRKCA	pp	NOS1
AKT1	pp	NOS3
CAV1	pp	NOS3
NOS3	pp	NOS3
PPP2R5A	pp	NOS3
SP1	pp	NOS3
DLL1	pp	NOTCH3
JAG1	pp	NOTCH3
JAG2	pp	NOTCH3
KAT2B	pp	NOTCH3
MAML1	pp	NOTCH3
PSEN1	pp	NOTCH3
SNW1	pp	NOTCH3
DLL1	pp	NOV
ESR1	pp	NR0B1
ESR2	pp	NR0B1
NCOR2	pp	NR1H4
PPARGC1A	pp	NR1H4
SMARCD3	pp	NR1H4
NR1I2	pp	NR1I2
PSMC5	pp	NR1I2
AR	pp	NR3C1
CREBBP	pp	NR3C1
ETS1	pp	NR3C1
ETS2	pp	NR3C1
GRIP1	pp	NR3C1
HNRNPU	pp	NR3C1
HSP90AA1	pp	NR3C1
JUN	pp	NR3C1
MAPK1	pp	NR3C1
MAPK8	pp	NR3C1
NCL	pp	NR3C1
NCOA1	pp	NR3C1
NCOR2	pp	NR3C1
NFKB1	pp	NR3C1
NR3C1	pp	NR3C1
PPARGC1A	pp	NR3C1
PRKACA	pp	NR3C1
PRKDC	pp	NR3C1
RAF1	pp	NR3C1

RELA	pp	NR3C1
SMAD3	pp	NR3C1
SMARCD1	pp	NR3C1
SUMO1	pp	NR3C1
SUMO4	pp	NR3C1
TBP	pp	NR3C1
TP53	pp	NR3C1
TSG101	pp	NR3C1
UBE2I	pp	NR3C1
RPS6KA1	pp	NR4A2
VHL	pp	NR4A2
MAGI3	pp	NRCAM
NRCAM	pp	NRCAM
MAFF	pp	NRF1
PPARGC1A	pp	NRF1
ADAM19	pp	NRG1
ERBB3	pp	NRG1
ERBB4	pp	NRG1
LIMK1	pp	NRG1
FGF2	pp	NRP1
GIPC1	pp	NRP1
NRP1	pp	NRP1
NRP2	pp	NRP1
NT5C2	pp	NT5C2
UBD	pp	NUB1
ATF3	pp	NUF2
PIM1	pp	NUMA1
TNKS	pp	NUMA1
THRA	pp	OASL
ODF1	pp	ODF1
SPAG4	pp	ODF1
RNF11	pp	OPTN
CD47	pp	P2RY2
ERO1L	pp	P4HB
P4HB	pp	P4HB
PTN	pp	P4HB
YWHAG	pp	P4HB
NDRG1	pp	PABPC1
PABPC1	pp	PABPC1
YWHAG	pp	PABPC1
DLST	pp	PAK4
FGFR2	pp	PAK4
PAK4	pp	PAK4
YWHAZ	pp	PAK4

ITGB5	pp	PALLD
IGFBP5	pp	PAPPA2
CTSG	pp	PARP1
GZMB	pp	PARP1
PARP1	pp	PARP1
PCNA	pp	PARP1
PRKDC	pp	PARP1
RBM14	pp	PARP1
RXRA	pp	PARP1
TP53	pp	PARP1
XRCC5	pp	PARP1
EWSR1	pp	PCBP1
HNRNPK	pp	PCBP1
LMNA	pp	PCBP1
PABPC1	pp	PCBP1
PABPC1	pp	PCBP2
PCBP1	pp	PCBP2
PCBP2	pp	PCBP2
PCBP1	pp	PCBP4
QKI	pp	PCBP4
RNF138	pp	PCBP4
PCCB	pp	PCCA
PCCB	pp	PCCB
CDC6	pp	PCNA
CDK6	pp	PCNA
CDKN1C	pp	PCNA
GADD45B	pp	PCNA
GADD45G	pp	PCNA
HDAC1	pp	PCNA
MCL1	pp	PCNA
MYBBP1A	pp	PCNA
PCNA	pp	PCNA
POLD2	pp	PCNA
PPP1CA	pp	PCNA
PRKDC	pp	PCNA
XRCC5	pp	PCNA
APOA1	pp	PDE1A
AKAP6	pp	PDE4D
PDE4D	pp	PDE4D
PDE4DIP	pp	PDE4D
PRKACA	pp	PDE4D
DUSP3	pp	PDGFA
FURIN	pp	PDGFA
PDAP1	pp	PDGFA

PDGFA pp PDGFA
PDGFRA pp PDGFA
SPARC pp PDGFA
CAV1 pp PDGFRA
ITGAV pp PDGFRA
PDGFRA pp PDGFRA
PIK3R1 pp PDGFRA
PTPN11 pp PDGFRA
SLC9A3R1 pp PDGFRA
STAT3 pp PDGFRA
DLAT pp PDHA1
PDHA1 pp PDHA1
PDK1 pp PDHA1
PDK2 pp PDHA1
PDK4 pp PDHA1
BACE1 pp PDIA3
SUMO4 pp PDIA3
DLAT pp PDK1
PDK2 pp PDK1
AKT1 pp PDK2
DLAT pp PDK2
HTT pp PDK2
PDK2 pp PDK2
DLAT pp PDK4
PDK4 pp PDK4
TRIM63 pp PDK4
STK35 pp PDLIM1
VIM pp PDLIM1
ADRBK1 pp PEBP1
CHUK pp PEBP1
IKBKB pp PEBP1
PEBP1 pp PEBP1
PRKCA pp PEBP1
PRKCB pp PEBP1
PRKCD pp PEBP1
PRKCG pp PEBP1
PRKCZ pp PEBP1
ITGB3 pp PECAM1
PECAM1 pp PECAM1
PRKCE pp PECAM1
PTPN11 pp PECAM1
XRCC6 pp PECAM1
HIF1A pp PER1
PER2 pp PER1

PER3	pp	PER1
CSNK1D	pp	PER2
PER3	pp	PER2
PF4	pp	PF4
PROC	pp	PF4
GBE1	pp	PGD
HIF1A	pp	PGK1
HSPH1	pp	PGK1
SUMO4	pp	PGK1
CCNA2	pp	PGR
CSNK2A1	pp	PGR
PGR	pp	PGR
XRCC6	pp	PGR
LPXN	pp	PHF21A
NFE2	pp	PHYHIP
PHYHIP	pp	PHYHIP
COIL	pp	PIAS4
NR4A2	pp	PIAS4
TICAM1	pp	PIAS4
PIK3CD	pp	PIK3CD
ABL1	pp	PIK3R1
ADAM12	pp	PIK3R1
AKT1	pp	PIK3R1
ANK3	pp	PIK3R1
ARAF	pp	PIK3R1
BCAR1	pp	PIK3R1
ERBB3	pp	PIK3R1
GAB1	pp	PIK3R1
LCK	pp	PIK3R1
MAPK8	pp	PIK3R1
MET	pp	PIK3R1
PECAM1	pp	PIK3R1
PIK3CD	pp	PIK3R1
PIK3R1	pp	PIK3R1
PTK2	pp	PIK3R1
PTPN11	pp	PIK3R1
RAC1	pp	PIK3R1
TEK	pp	PIK3R1
TGFBR1	pp	PIK3R1
TYRO3	pp	PIK3R1
YWHAG	pp	PIK3R1
ARAF	pp	PKM2
NDRG1	pp	PKM2
PKM2	pp	PKM2

TRIM63	pp	PKM2
CASP3	pp	PKN1
CD44	pp	PKN1
PKN1	pp	PKN1
CASP3	pp	PKN2
MAP3K2	pp	PKN2
PLA2G2A	pp	PLA2G2A
SLC9A3R1	pp	PLCB2
EGFR	pp	PLD2
F10	pp	PLG
IGF2	pp	PLG
IGFBP3	pp	PLG
KLK3	pp	PLG
LAMA3	pp	PLG
MMP7	pp	PLG
PLG	pp	PLG
SERPINF2	pp	PLG
SPARC	pp	PLG
THBS1	pp	PLG
PLOD2	pp	PLOD2
DHX37	pp	PLXNA2
FNBP4	pp	PLXNA2
HACE1	pp	PLXNA2
IFT80	pp	PLXNA2
KIAA1199	pp	PLXNA2
UBAP2L	pp	PLXNA2
ATP5C1	pp	POLA2
DDX24	pp	POLA2
FGFR3	pp	POLA2
IGFBP3	pp	POLA2
CDK2	pp	POLD2
POLR2C	pp	POLR2C
STC2	pp	POLR2C
KAT5	pp	POLR3F
STC2	pp	POLR3F
FOXD3	pp	POU5F1
MNAT1	pp	POU5F1
SOX10	pp	POU5F1
SOX2	pp	POU5F1
SOX8	pp	POU5F1
GADD45B	pp	PPARA
GADD45G	pp	PPARA
MAPK1	pp	PPARA
MAPK3	pp	PPARA

MED1	pp	PPARA
NCOA1	pp	PPARA
NCOR2	pp	PPARA
PPARA	pp	PPARA
PPARGC1A	pp	PPARA
RXRA	pp	PPARA
CNOT1	pp	PPARG
GADD45B	pp	PPARG
GADD45G	pp	PPARG
MAPK1	pp	PPARG
MAPK8	pp	PPARG
MED1	pp	PPARG
NCOR2	pp	PPARG
NR0B1	pp	PPARG
PPARG	pp	PPARG
PPARGC1A	pp	PPARG
RXRB	pp	PPARG
SMARCD3	pp	PPARG
ESR2	pp	PPARGC1A
ESRRA	pp	PPARGC1A
HNF4A	pp	PPARGC1A
MAPK14	pp	PPARGC1A
MYBBP1A	pp	PPARGC1A
NR1I2	pp	PPARGC1A
NR1I3	pp	PPARGC1A
THRB	pp	PPARGC1A
EFNA1	pp	PPBP
PPBP	pp	PPBP
CAST	pp	PPFIA4
PPHLN1	pp	PPHLN1
CDC5L	pp	PPP1CA
MAPK1	pp	PPP1CA
MAPK3	pp	PPP1CA
PPP1CA	pp	PPP1CA
PPP1R8	pp	PPP1CA
RB1	pp	PPP1CA
SPRED1	pp	PPP1CA
TP53	pp	PPP1CA
PRKCA	pp	PPP1R14A
PRKCE	pp	PPP1R14A
PRKCZ	pp	PPP1R14A
PRKD1	pp	PPP1R14A
CDC5L	pp	PPP1R8
CSNK2A1	pp	PPP1R8

CSNK2A2	pp	PPP1R8
HDAC2	pp	PPP1R8
LYN	pp	PPP1R8
PRKACA	pp	PPP1R8
BMPR1B	pp	PPP2CA
DVL3	pp	PPP2CA
MAPK1	pp	PPP2CA
MAPK3	pp	PPP2CA
PPP1CA	pp	PPP2CA
PPP2R1A	pp	PPP2CA
PPP2R1B	pp	PPP2CA
PPP2R2A	pp	PPP2CA
PPP2R5B	pp	PPP2CA
PPP2R5E	pp	PPP2CA
PTN	pp	PPP2CA
RELA	pp	PPP2CA
RPS6KB1	pp	PPP2CA
SGOL1	pp	PPP2CA
SGOL1	pp	PPP2CB
SGOL1	pp	PPP2R1A
SMAD2	pp	PPP2R1A
AKAP9	pp	PPP2R1B
PPP2R2A	pp	PPP2R1B
PPP2R5D	pp	PPP2R1B
SGOL1	pp	PPP2R1B
NDRG1	pp	PPP2R2A
PPP2R1A	pp	PPP2R2A
PPP2R1A	pp	PPP2R5A
SGOL1	pp	PPP2R5A
IER3	pp	PPP2R5B
SGOL1	pp	PPP2R5B
IER3	pp	PPP2R5C
PPP2CA	pp	PPP2R5C
PPP2R1B	pp	PPP2R5C
SGOL1	pp	PPP2R5C
SGOL1	pp	PPP2R5D
PPP2R1B	pp	PPP2R5E
SGOL1	pp	PPP2R5E
AKAP5	pp	PPP3CA
ATP2B4	pp	PPP3CA
BCL2	pp	PPP3CA
CABIN1	pp	PPP3CA
CASP3	pp	PPP3CA
CRTC2	pp	PPP3CA

FKBP1A	pp	PPP3CA
FKBP8	pp	PPP3CA
GABRA1	pp	PPP3CA
GABRB2	pp	PPP3CA
GABRG2	pp	PPP3CA
MYOZ2	pp	PPP3CA
MYOZ3	pp	PPP3CA
PPP3CA	pp	PPP3CA
PPP3R1	pp	PPP3CA
PRKCZ	pp	PPP3CA
RCAN2	pp	PPP3CA
SOD1	pp	PPP3CA
SYNJ1	pp	PPP3CA
BCL2	pp	PRKACA
ESR1	pp	PRKACA
SLC9A3R2	pp	PRKACA
VIM	pp	PRKACA
AKAP12	pp	PRKAR2A
AKAP5	pp	PRKAR2A
AKAP6	pp	PRKAR2A
AKAP9	pp	PRKAR2A
CBFA2T3	pp	PRKAR2A
CFTR	pp	PRKAR2A
PRKAR2A	pp	PRKAR2A
AKAP12	pp	PRKCA
AKAP5	pp	PRKCA
C1QBP	pp	PRKCA
DLG4	pp	PRKCA
FAS	pp	PRKCA
FLNA	pp	PRKCA
GNB2L1	pp	PRKCA
HSP90AA1	pp	PRKCA
PLD2	pp	PRKCA
PPARA	pp	PRKCA
PRKCA	pp	PRKCA
PRKCZ	pp	PRKCA
RAC1	pp	PRKCA
YWHAG	pp	PRKCA
YWHAZ	pp	PRKCA
BMPR2	pp	PRKCB
GNB2L1	pp	PRKCB
GSK3B	pp	PRKCB
KIT	pp	PRKCB
PPP2CB	pp	PRKCB

PRKCB	pp	PRKCB
YWHAG	pp	PRKCB
ADAM9	pp	PRKCD
C1QBP	pp	PRKCD
ESRRA	pp	PRKCD
GNB2L1	pp	PRKCD
HSP90AA1	pp	PRKCD
IGF1R	pp	PRKCD
LCK	pp	PRKCD
LYN	pp	PRKCD
MAPK1	pp	PRKCD
MAPK3	pp	PRKCD
PLD2	pp	PRKCD
PPARA	pp	PRKCD
PPP1CA	pp	PRKCD
PPP2CA	pp	PRKCD
PPP2R5C	pp	PRKCD
PRKCD	pp	PRKCD
PRKCZ	pp	PRKCD
PRKDC	pp	PRKCD
RAC1	pp	PRKCD
RAF1	pp	PRKCD
RUNX2	pp	PRKCD
YWHAG	pp	PRKCD
AKAP9	pp	PRKCE
CFTR	pp	PRKCE
COPB2	pp	PRKCE
GNB2L1	pp	PRKCE
HK2	pp	PRKCE
MAPK3	pp	PRKCE
PRKCE	pp	PRKCE
PRKD1	pp	PRKCE
PPP1R14A	pp	PRKCG
YWHAB	pp	PRKCG
YWHAG	pp	PRKCG
C1QBP	pp	PRKCZ
CASP3	pp	PRKCZ
DAPK3	pp	PRKCZ
FADD	pp	PRKCZ
MAP2K1	pp	PRKCZ
MAP2K5	pp	PRKCZ
MAPK1	pp	PRKCZ
MAPK3	pp	PRKCZ
PRKCZ	pp	PRKCZ

RHOA	pp	PRKCZ
TRAF6	pp	PRKCZ
ABL1	pp	PRKD1
EGFR	pp	PRKD1
GNB2L1	pp	PRKD1
IGF1R	pp	PRKD1
PRKD1	pp	PRKD1
AKT1	pp	PRKDC
CASP3	pp	PRKDC
GZMB	pp	PRKDC
MAPK8	pp	PRKDC
PRKDC	pp	PRKDC
SGOL1	pp	PRKDC
SP1	pp	PRKDC
XRCC5	pp	PRKDC
YWHAG	pp	PRKDC
AR	pp	PRMT1
ESR1	pp	PRMT1
ILF3	pp	PRMT1
PRMT1	pp	PRMT1
QKI	pp	PRMT1
YWHAG	pp	PRMT1
F2	pp	PROC
F8	pp	PROC
PROC	pp	PROC
PROS1	pp	PROC
C4BPB	pp	PROS1
F10	pp	PROS1
F2	pp	PROS1
F5	pp	PROS1
F8	pp	PROS1
TYRO3	pp	PROS1
MAFF	pp	PRRX1
SOX10	pp	PRRX1
SRF	pp	PRRX1
ACTG1	pp	PRSS23
EFNA3	pp	PRSS23
GRK6	pp	PRSS23
BACE1	pp	PSAP
PSAP	pp	PSAP
SMAD2	pp	PSAP
BACE1	pp	PSEN1
BCL2	pp	PSEN1
BCL2L1	pp	PSEN1

CASP3	pp	PSEN1
CDK5	pp	PSEN1
CTNNA1	pp	PSEN1
CTNNB1	pp	PSEN1
DLL1	pp	PSEN1
FBXW7	pp	PSEN1
FLNA	pp	PSEN1
GSK3B	pp	PSEN1
PIK3R1	pp	PSEN1
PRKACA	pp	PSEN1
PRKCZ	pp	PSEN1
PSEN1	pp	PSEN1
UBQLN1	pp	PSEN1
CASP3	pp	PSEN2
CSNK1D	pp	PSEN2
CSNK2A1	pp	PSEN2
CTNNB1	pp	PSEN2
NOTCH3	pp	PSEN2
UBQLN1	pp	PSEN2
CSNK2A1	pp	PSMA3
NDRG1	pp	PSMC2
PSMD2	pp	PSMC2
SKIL	pp	PSMC2
SUMO4	pp	PSMC2
TBP	pp	PSMC2
NDRG1	pp	PSMC3
PSMC3	pp	PSMC3
PSMC5	pp	PSMC3
VHL	pp	PSMC3
ESR1	pp	PSMC5
THRB	pp	PSMC5
NDRG1	pp	PSMD2
PSMD2	pp	PSMD2
PTN	pp	PSMD2
CCDC85B	pp	PSMF1
QKI	pp	PSMF1
TRAF2	pp	PSMF1
CASP3	pp	PTBP1
PCBP1	pp	PTBP1
PCBP2	pp	PTBP1
PRKACA	pp	PTBP1
PTBP1	pp	PTBP1
PTBP2	pp	PTBP2
QKI	pp	PTBP2

HK2 pp PTGDS
BAT5 pp PTGS2
CAV1 pp PTGS2
NUCB1 pp PTGS2
PTGS2 pp PTGS2
CD47 pp PTK2
CSPG4 pp PTK2
EPHB2 pp PTK2
FLT4 pp PTK2
GIT1 pp PTK2
GSK3B pp PTK2
GZMB pp PTK2
LCK pp PTK2
LPXN pp PTK2
PTK2 pp PTK2
ATP5C1 pp PTN
GADD45G pp PTN
GSTP1 pp PTN
KPNB1 pp PTN
MLLT3 pp PTN
PIAS4 pp PTN
PTN pp PTN
CAV1 pp PTPN11
CDH5 pp PTPN11
EPHA2 pp PTPN11
ERBB2 pp PTPN11
FLT4 pp PTPN11
MAP3K4 pp PTPN11
PRKCA pp PTPN11
PRKCB pp PTPN11
PTK2 pp PTPN11
CDH5 pp PTPRB
CNTN1 pp PTPRB
CNTNAP1 pp PTPRB
F11R pp PTPRB
MAGI3 pp PTPRB
NRCAM pp PTPRB
TEK pp PTPRB
TNC pp PTPRB
CSNK2A1 pp PTPRC
CSNK2A2 pp PTPRC
PTPRC pp PTPRC
RNF11 pp PTPRC
DAZ4 pp QKI

FUBP3 pp QKI
HCLS1 pp QKI
HNRNPK pp QKI
PCBP1 pp QKI
PCBP2 pp QKI
PTBP1 pp QKI
CDKN1A pp RAB1A
RAB5A pp RAB5A
SDCBP pp RAB5A
TBC1D3C pp RAB5A
TBC1D3F pp RAB5A
AKT1 pp RAC1
ARHGDIB pp RAC1
CASP3 pp RAC1
DVL1 pp RAC1
RAC1 pp RAC1
RPS6KB1 pp RAC1
SET pp RAC1
TBC1D3F pp RAC1
AGTR1 pp RAF1
AKT1 pp RAF1
AR pp RAF1
CPS1 pp RAF1
IL2RB pp RAF1
MAP3K5 pp RAF1
MAPK1 pp RAF1
MAPK3 pp RAF1
MYC pp RAF1
PEBP1 pp RAF1
PKM2 pp RAF1
PRKACA pp RAF1
PRKCA pp RAF1
PRKCE pp RAF1
PRKCZ pp RAF1
RAF1 pp RAF1
RB1 pp RAF1
TSC22D3 pp RAF1
YWHAB pp RAF1
YWHAE pp RAF1
YWHAG pp RAF1
YWHAZ pp RAF1
ABL1 pp RB1
AR pp RB1
CASP10 pp RB1

CASP3	pp	RB1
CASP9	pp	RB1
CCNA1	pp	RB1
CCND2	pp	RB1
CDK1	pp	RB1
CDK2	pp	RB1
CDK4	pp	RB1
HDAC1	pp	RB1
MAPK1	pp	RB1
MNAT1	pp	RB1
MYC	pp	RB1
PIK3R1	pp	RB1
PRKCB	pp	RB1
RB1	pp	RB1
SNW1	pp	RB1
SP1	pp	RB1
TAF1	pp	RB1
TGM2	pp	RB1
QKI	pp	RBM14
ATXN1	pp	RBM9
QKI	pp	RBM9
RBPMS	pp	RBM9
HMGB1	pp	RBPJ
KAT2B	pp	RBPJ
NCOR2	pp	RBPJ
NOTCH3	pp	RBPJ
SNW1	pp	RBPJ
ATXN1	pp	RBPMS
EWSR1	pp	RBPMS
QKI	pp	RBPMS
RBPMS	pp	RBPMS
SERF2	pp	RBPMS
GSK3B	pp	RCAN1
MAPK3	pp	RCAN1
PPP3CA	pp	RCAN1
RCBTB2	pp	RCBTB2
KDM1A	pp	RCOR1
PHF21A	pp	RCOR1
L1CAM	pp	RDX
ROCK1	pp	RDX
SLC9A3R1	pp	RDX
SLC9A3R2	pp	RDX
AES	pp	RELA
AR	pp	RELA

CHUK	pp	RELA
CREBBP	pp	RELA
CSNK2A1	pp	RELA
DDX1	pp	RELA
ESR1	pp	RELA
FOS	pp	RELA
HDAC1	pp	RELA
HDAC2	pp	RELA
IKBKB	pp	RELA
JUN	pp	RELA
KAT2B	pp	RELA
MYC	pp	RELA
NCOR2	pp	RELA
NFKB1	pp	RELA
PARP1	pp	RELA
PGR	pp	RELA
PPARA	pp	RELA
PPP2R1B	pp	RELA
PRKACA	pp	RELA
PRKCZ	pp	RELA
RELA	pp	RELA
RXRA	pp	RELA
SIN3A	pp	RELA
SIRT1	pp	RELA
SP1	pp	RELA
SRF	pp	RELA
TAF1	pp	RELA
TBP	pp	RELA
TGM2	pp	RELA
TSC22D3	pp	RELA
ARHGDIB	pp	RHOA
CAV1	pp	RHOA
CNTNAP1	pp	RHOA
FLNA	pp	RHOA
PKN1	pp	RHOA
PKN2	pp	RHOA
PRKACA	pp	RHOA
PRKCA	pp	RHOA
RHOA	pp	RHOA
ROCK1	pp	RHOA
SMURF1	pp	RHOA
TGM2	pp	RHOA
BNIP3L	pp	RINT1
CCDC50	pp	RIPK1

PTK2	pp	RIPK1
RIPK1	pp	RIPK1
RNF11	pp	RIPK1
TNFAIP3	pp	RIPK1
QKI	pp	RNF138
CSNK2A1	pp	RNF7
CSNK2B	pp	RNF7
CASP3	pp	ROCK1
PTK2	pp	ROCK1
ROCK1	pp	ROCK1
SMAD2	pp	ROCK1
TSG101	pp	ROCK1
SGOL1	pp	RPL10A
NDRG1	pp	RPL24
SGOL1	pp	RPL28
SMAD4	pp	RPL28
NDRG1	pp	RPL3
NDRG1	pp	RPL4
RPL7	pp	RPL7
SGOL1	pp	RPL7
SGOL1	pp	RPL7A
PTN	pp	RPLP1
RPLP1	pp	RPLP1
SGOL1	pp	RPLP1
XRCC6	pp	RPLP1
NDRG1	pp	RPN2
NDRG1	pp	RPS16
NDRG1	pp	RPS20
NDRG1	pp	RPS26
NDRG1	pp	RPS3
RELA	pp	RPS3
NDRG1	pp	RPS6
RPS6KB1	pp	RPS6
MAPK1	pp	RPS6KA1
MAPK3	pp	RPS6KA1
MITF	pp	RPS6KA1
RPS6KA1	pp	RPS6KA1
MAP2K1	pp	RPS6KA4
MAPK14	pp	RPS6KA4
RPS6KA4	pp	RPS6KA4
CDK1	pp	RPS6KB1
MAPK3	pp	RPS6KB1
MAPK8	pp	RPS6KB1
PDK1	pp	RPS6KB1

NDRG1	pp	RPS8
CBFA2T2	pp	RUNX1
CBFA2T3	pp	RUNX1
FOS	pp	RUNX1
JUN	pp	RUNX1
TLE1	pp	RUNX1
TLE2	pp	RUNX1
CBFA2T3	pp	RUNX1T1
HSP90AA1	pp	RUNX1T1
ID3	pp	RUNX1T1
KPNB1	pp	RUNX1T1
NCOR2	pp	RUNX1T1
PRKAR2A	pp	RUNX1T1
RUNX1T1	pp	RUNX1T1
SIN3A	pp	RUNX1T1
ZNF652	pp	RUNX1T1
AR	pp	RUNX2
CDK1	pp	RUNX2
RB1	pp	RUNX2
SMAD2	pp	RUNX2
SMAD3	pp	RUNX2
XRCC5	pp	RUNX2
XRCC6	pp	RUNX2
BCL3	pp	RUVBL2
CTNNB1	pp	RUVBL2
HDAC1	pp	RUVBL2
NDRG1	pp	RUVBL2
ACVR1	pp	RXRA
CNOT1	pp	RXRA
CTNNB1	pp	RXRA
ESR1	pp	RXRA
GADD45G	pp	RXRA
MAPK1	pp	RXRA
MED1	pp	RXRA
NCOA1	pp	RXRA
NCOR2	pp	RXRA
NR1H4	pp	RXRA
NR1I2	pp	RXRA
NR1I3	pp	RXRA
NR4A2	pp	RXRA
PPARG	pp	RXRA
PPARGC1A	pp	RXRA
PSMC5	pp	RXRA
SMAD2	pp	RXRA

SMARCD3 pp RXRA
SP1 pp RXRA
SRF pp RXRA
TBP pp RXRA
THRA pp RXRA
THRB pp RXRA
NCOA1 pp RXRB
NR1I2 pp RXRB
NR4A2 pp RXRB
RXRB pp RXRB
THRA pp RXRB
THRB pp RXRB
COPS5 pp S100A7
EGFR pp S100A7
S100A7 pp S100A7
TGM2 pp S100A7
NDRG1 pp S100B
S100B pp S100B
TNIP1 pp S100B
TP53 pp S100B
S100P pp S100P
INSIG2 pp SCAP
SCAP pp SCAP
SCHIP1 pp SCHIP1
ANK3 pp SCN1B
CNTN1 pp SCNN1B
CASK pp SDC2
EPHB2 pp SDC2
FGF2 pp SDC2
KAL1 pp SDC2
LAMA3 pp SDC2
NF1 pp SDC2
PF4 pp SDC2
PRKCA pp SDC2
PRKCB pp SDC2
PRKCG pp SDC2
REG3A pp SDC2
SDCBP pp SDC2
SPARC pp SDC2
TNFSF13 pp SDC2
TRAPPC4 pp SDC2
EPHB2 pp SDCBP
NF2 pp SDCBP
RNF11 pp SDCBP

SDCBP	pp	SDCBP
DCTN1	pp	SEC23A
NDRG1	pp	SEC23A
SELP	pp	SELP
NRP1	pp	SEMA3F
NRP2	pp	SEMA3F
QKI	pp	SERF2
F2	pp	SERPINC1
PLG	pp	SERPINC1
SDC2	pp	SERPINC1
SERPINC1	pp	SERPINC1
SERPINF2	pp	SERPINF2
TGM2	pp	SERPINF2
PPP2CA	pp	SET
PPP2R1B	pp	SET
SET	pp	SET
SGOL1	pp	SET
C1QBP	pp	SFRS1
PPARGC1A	pp	SFRS4
PPARGC1A	pp	SFRS5
PPARGC1A	pp	SFRS6
SFRS4	pp	SFRS6
SFTPD	pp	SFTPD
SGOL1	pp	SGOL1
CA12	pp	SGSM2
EGFR	pp	SGSM2
PTN	pp	SGSM2
MAP3K2	pp	SH2D2A
A2M	pp	SHBG
ACTB	pp	SHBG
COL1A2	pp	SHBG
CTSD	pp	SHBG
FLNA	pp	SHBG
PKM2	pp	SHBG
PTGDS	pp	SHBG
SEMA3F	pp	SHBG
SHBG	pp	SHBG
NDRG1	pp	SHMT2
SHMT2	pp	SHMT2
AFF1	pp	SIAH1
GAPDH	pp	SIAH1
SIAH1	pp	SIAH1
SLC9A3R2	pp	SIAH1
AFF1	pp	SIAH2

ENO2 pp SIGIRR
SIGIRR pp SIGIRR
HCFC1 pp SIN3A
MXI1 pp SIN3A
HDAC1 pp SIN3B
MXI1 pp SIN3B
HNF4A pp SIRT1
PPARGC1A pp SIRT1
FN1 pp SKIL
LRP1 pp SKIL
PIAS4 pp SKIL
SKIL pp SKIL
SMAD2 pp SKIL
SMAD3 pp SKIL
SMAD4 pp SKIL
XRCC6 pp SKIL
CDKN1A pp SLC25A11
SLC25A11 pp SLC25A11
LNPEP pp SLC2A4
UBE2I pp SLC2A4
EGFR pp SLC3A2
ICAM1 pp SLC3A2
SLC7A11 pp SLC3A2
EGFR pp SLC9A3R1
GRK6 pp SLC9A3R1
NF2 pp SLC9A3R1
TBC1D10A pp SLC9A3R1
ADORA2B pp SLC9A3R2
TBC1D10A pp SLC9A3R2
AKT1 pp SMAD2
ARL4D pp SMAD2
CDK4 pp SMAD2
CREBBP pp SMAD2
MAPK1 pp SMAD2
MAPK3 pp SMAD2
MAPK8 pp SMAD2
OS9 pp SMAD2
PIAS4 pp SMAD2
SMAD2 pp SMAD2
SMAD3 pp SMAD2
SNW1 pp SMAD2
TGFBR1 pp SMAD2
TP53 pp SMAD2
AKT1 pp SMAD3

ATF3	pp	SMAD3
CDK2	pp	SMAD3
CDK4	pp	SMAD3
CREBBP	pp	SMAD3
DVL1	pp	SMAD3
ESR1	pp	SMAD3
ESR2	pp	SMAD3
ETS1	pp	SMAD3
FOS	pp	SMAD3
JUND	pp	SMAD3
LMNA	pp	SMAD3
MAP3K7	pp	SMAD3
MAPK1	pp	SMAD3
MAPK14	pp	SMAD3
MBD1	pp	SMAD3
PIAS4	pp	SMAD3
PPP2R1A	pp	SMAD3
PRKAR2A	pp	SMAD3
SMAD3	pp	SMAD3
SP1	pp	SMAD3
TP53	pp	SMAD3
TP73	pp	SMAD3
XRCC6	pp	SMAD3
AKT1	pp	SMAD4
ATF7IP	pp	SMAD4
COPS5	pp	SMAD4
CREBBP	pp	SMAD4
DVL1	pp	SMAD4
ESR1	pp	SMAD4
EWSR1	pp	SMAD4
FN1	pp	SMAD4
MAPK1	pp	SMAD4
PAPPA2	pp	SMAD4
PIAS4	pp	SMAD4
PRKAR2A	pp	SMAD4
SMAD2	pp	SMAD4
SMAD3	pp	SMAD4
SMAD4	pp	SMAD4
SP1	pp	SMAD4
UBE2I	pp	SMAD4
AKT1	pp	SMAD7
BMPR1B	pp	SMAD7
CTNNB1	pp	SMAD7
DVL1	pp	SMAD7

MBD1	pp	SMAD7
PIAS4	pp	SMAD7
SMAD7	pp	SMAD7
SMURF1	pp	SMAD7
XRCC6	pp	SMAD7
ACTB	pp	SMAD9
AFF1	pp	SMAD9
EIF3E	pp	SMAD9
FN1	pp	SMAD9
KIAA0226	pp	SMAD9
MBD1	pp	SMAD9
PPP2R5E	pp	SMAD9
PSAP	pp	SMAD9
SMAD2	pp	SMAD9
SMAD3	pp	SMAD9
SMAD4	pp	SMAD9
TERF1	pp	SMAD9
UBQLN1	pp	SMAD9
UBQLN4	pp	SMAD9
YWHAQ	pp	SMAD9
TNIP2	pp	SMARCD1
TP53	pp	SMARCD1
ARL4D	pp	SMURF1
CDK14	pp	SMURF1
CSNK2A2	pp	SMURF1
SFRS4	pp	SMURF1
SFRS5	pp	SMURF1
SMAD3	pp	SMURF1
CSNK1D	pp	SNAPIN
KAT5	pp	SNAPIN
LAMC1	pp	SNAPIN
MET	pp	SNAPIN
PRKACA	pp	SNAPIN
SHBG	pp	SNAPIN
SMAD2	pp	SNAPIN
ADRBK1	pp	SNCA
BAD	pp	SNCA
BCL2L1	pp	SNCA
CSNK1D	pp	SNCA
CSNK2A1	pp	SNCA
CSNK2A2	pp	SNCA
ELK1	pp	SNCA
GRK5	pp	SNCA
GRK6	pp	SNCA

LYN	pp	SNCA
MAPK1	pp	SNCA
PRKCE	pp	SNCA
YWHAB	pp	SNCA
YWHAE	pp	SNCA
YWHAH	pp	SNCA
ENO2	pp	SNUPN
HDAC2	pp	SNW1
NR0B1	pp	SNW1
PGR	pp	SNW1
SMAD3	pp	SNW1
BCL2	pp	SOD1
SOD1	pp	SOD1
SOSTDC1	pp	SOSTDC1
SOX10	pp	SOX10
ATF7IP	pp	SP1
CASP3	pp	SP1
CSNK2A1	pp	SP1
ESR1	pp	SP1
ESR2	pp	SP1
ETS1	pp	SP1
HCFC1	pp	SP1
HDAC1	pp	SP1
HDAC2	pp	SP1
HIF1A	pp	SP1
HNF4A	pp	SP1
MAPK1	pp	SP1
MAPK3	pp	SP1
MYC	pp	SP1
PER3	pp	SP1
PSMC5	pp	SP1
SMAD2	pp	SP1
SOX10	pp	SP1
SOX8	pp	SP1
SP1	pp	SP1
SREBF2	pp	SP1
TP53	pp	SP1
YY1	pp	SP1
SPAG4	pp	SPAG4
SPARC	pp	SPARC
TGFB1	pp	SPARC
TGM2	pp	SPARC
THBS1	pp	SPARC
VEGFA	pp	SPARC

CTRC	pp	SPINK1
NRSN1	pp	SPINK1
PRSS7	pp	SPINK1
CSNK2A1	pp	SPP1
CSNK2A2	pp	SPP1
F2	pp	SPP1
ITGAV	pp	SPP1
MMP3	pp	SPP1
MMP7	pp	SPP1
PRKCA	pp	SPP1
ATXN1	pp	SREBF1
ESR1	pp	SREBF1
ID3	pp	SREBF1
MAPK1	pp	SREBF1
MAPK3	pp	SREBF1
NR0B1	pp	SREBF1
SMARCD3	pp	SREBF1
SP1	pp	SREBF1
SREBF1	pp	SREBF1
SUMO1	pp	SREBF1
TWIST2	pp	SREBF1
UBE2I	pp	SREBF1
YY1	pp	SREBF1
CASP3	pp	SREBF2
INSIG2	pp	SREBF2
KPNB1	pp	SREBF2
MAPK3	pp	SREBF2
SCAP	pp	SREBF2
SMAD3	pp	SREBF2
SREBF2	pp	SREBF2
SUMO1	pp	SREBF2
UBE2I	pp	SREBF2
CASP3	pp	SRF
CREBBP	pp	SRF
CSNK2A1	pp	SRF
GATA4	pp	SRF
NCOA1	pp	SRF
NCOR2	pp	SRF
PRKDC	pp	SRF
RPS6KA1	pp	SRF
SRF	pp	SRF
ENO2	pp	ST3GAL2
AKT1	pp	STAT1
EGFR	pp	STAT1

FGFR3	pp	STAT1
FOS	pp	STAT1
KIT	pp	STAT1
LCK	pp	STAT1
PDGFRA	pp	STAT1
PRKCD	pp	STAT1
PRMT1	pp	STAT1
PTK2	pp	STAT1
PTPN11	pp	STAT1
RAC1	pp	STAT1
RELA	pp	STAT1
STAT1	pp	STAT1
SUMO4	pp	STAT1
UBE2I	pp	STAT1
AR	pp	STAT3
CCND1	pp	STAT3
CDKN1A	pp	STAT3
CREBBP	pp	STAT3
EGFR	pp	STAT3
EPHA3	pp	STAT3
FGFR3	pp	STAT3
GNB2L1	pp	STAT3
HDAC1	pp	STAT3
HDAC2	pp	STAT3
HNF1A	pp	STAT3
HSP90AA1	pp	STAT3
IL2RB	pp	STAT3
JUN	pp	STAT3
KAT5	pp	STAT3
LCK	pp	STAT3
MAP3K7	pp	STAT3
MAPK1	pp	STAT3
MAPK3	pp	STAT3
MAPK8	pp	STAT3
MET	pp	STAT3
NCOA1	pp	STAT3
NFKB1	pp	STAT3
NR3C1	pp	STAT3
PDIA3	pp	STAT3
PRKCD	pp	STAT3
PTPN11	pp	STAT3
RAC1	pp	STAT3
RELA	pp	STAT3
STAT1	pp	STAT3

STAT3	pp	STAT3
SUMO4	pp	STAT3
CREBBP	pp	STAT4
MAP2K6	pp	STAT4
MAPK14	pp	STAT4
STAT3	pp	STAT4
STAT4	pp	STAT4
ADAM11	pp	STC2
ARRB2	pp	STC2
PEBP1	pp	STC2
PPP1R8	pp	STC2
PTN	pp	STC2
BMPR1B	pp	STK35
SMAD4	pp	STK35
SMURF1	pp	STK35
STK35	pp	STK35
HTT	pp	SUMO1
SOX10	pp	SUMO1
TP73	pp	SUMO1
UBE2I	pp	SUMO1
PIAS4	pp	SUMO2
SOX10	pp	SUMO2
SUMO2	pp	SUMO2
TRIM63	pp	SUMO2
UBE2I	pp	SUMO2
VIM	pp	SUMO2
HSF1	pp	SUMO3
PIAS4	pp	SUMO3
SOX10	pp	SUMO3
UBE2I	pp	SUMO3
VIM	pp	SUMO3
FNBP4	pp	SYNJ1
TAF1	pp	TAF1
TBP	pp	TAF1
PRKCD	pp	TAGLN
NDRG1	pp	TARS
RIPK1	pp	TAX1BP1
RNF11	pp	TAX1BP1
TAX1BP1	pp	TAX1BP1
TBC1D10A	pp	TBC1D10A
DR1	pp	TBP
HNRNPK	pp	TBP
MYC	pp	TBP
NCOA1	pp	TBP

RB1	pp	TBP
SP1	pp	TBP
TBP	pp	TBP
YWHAE	pp	TBP
TBX3	pp	TBX3
ANGPT1	pp	TEK
PTPN11	pp	TEK
TEK	pp	TEK
TNIP2	pp	TEK
KPNB1	pp	TERF1
POT1	pp	TERF1
TERF1	pp	TERF1
TNKS	pp	TERF1
CDKN1A	pp	TEX11
UBE2I	pp	TFAP2B
DPT	pp	TGFB1
ITGAV	pp	TGFB1
ITGB8	pp	TGFB1
SDC2	pp	TGFB1
TGFB1	pp	TGFB1
TGFB2	pp	TGFB1
TGFBR1	pp	TGFB1
TGFBR2	pp	TGFB1
TGFBR3	pp	TGFB1
YWHAE	pp	TGFB1
TGFB2	pp	TGFB2
TGFB3	pp	TGFB2
TGFBR3	pp	TGFB2
TGFBR3	pp	TGFB3
ACVR1	pp	TGFBR1
ARL4D	pp	TGFBR1
BMPR1B	pp	TGFBR1
CAV1	pp	TGFBR1
CDK14	pp	TGFBR1
CDK4	pp	TGFBR1
CDK6	pp	TGFBR1
CHUK	pp	TGFBR1
CSNK2A2	pp	TGFBR1
IKBKB	pp	TGFBR1
MAP3K7	pp	TGFBR1
PPP2R2A	pp	TGFBR1
RHOA	pp	TGFBR1
SMAD3	pp	TGFBR1
SMAD4	pp	TGFBR1

SMAD7	pp	TGFBR1
STK35	pp	TGFBR1
TGFB2	pp	TGFBR1
TGFB3	pp	TGFBR1
TGFBR1	pp	TGFBR1
TGFBR2	pp	TGFBR1
XIAP	pp	TGFBR1
CCNB2	pp	TGFBR2
CDK1	pp	TGFBR2
CTNNB1	pp	TGFBR2
PIK3R1	pp	TGFBR2
SMAD3	pp	TGFBR2
SMAD4	pp	TGFBR2
SMAD7	pp	TGFBR2
TGFB2	pp	TGFBR2
TGFB3	pp	TGFBR2
TGFBR2	pp	TGFBR2
TGFBR3	pp	TGFBR2
GIPC1	pp	TGFBR3
SDC2	pp	TGFBR3
CASP3	pp	TGM2
EIF5A	pp	TGM2
HIST1H2BG	pp	TGM2
HIST2H2BE	pp	TGM2
KPNA3	pp	TGM2
KPNA4	pp	TGM2
MAP3K12	pp	TGM2
PLCD1	pp	TGM2
PPHLN1	pp	TGM2
SMAD2	pp	TGM2
SMAD3	pp	TGM2
TGM2	pp	TGM2
TMSB4X	pp	TGM2
TUBB	pp	TGM2
QKI	pp	THAP1
THAP1	pp	THAP1
CD47	pp	THBS1
JAG1	pp	THBS1
TGFB1	pp	THBS1
ESR1	pp	THRA
NCOA1	pp	THRA
ACVR1	pp	THRB
CNOT1	pp	THRB
ESR1	pp	THRB

NCOA1	pp	THRB
THRB	pp	THRB
THY1	pp	THY1
RIPK1	pp	TICAM1
TNFAIP3	pp	TICAM1
TRAF3	pp	TICAM1
CDK1	pp	TK1
GAPDH	pp	TK1
PRDM1	pp	TLE1
TK1	pp	TLE1
TLE1	pp	TLE1
TLE2	pp	TLE1
PRDM1	pp	TLE2
NDRG1	pp	TLE3
BNIP3	pp	TMEM11
BNIP3L	pp	TMEM11
SEMA3F	pp	TMEM22
ILK	pp	TMSB4X
CNTN1	pp	TNC
ADAM9	pp	TNF
TNF	pp	TNF
TRAF2	pp	TNF
CCDC50	pp	TNFAIP3
CHUK	pp	TNFAIP3
IKBKB	pp	TNFAIP3
IKBKG	pp	TNFAIP3
LAPTM5	pp	TNFAIP3
RNF11	pp	TNFAIP3
TAX1BP1	pp	TNFAIP3
TNFAIP3	pp	TNFAIP3
TNIP1	pp	TNFAIP3
TNIP2	pp	TNFAIP3
TRAF1	pp	TNFAIP3
TRAF2	pp	TNFAIP3
TRAF6	pp	TNFAIP3
YWHAB	pp	TNFAIP3
YWHAE	pp	TNFAIP3
YWHAG	pp	TNFAIP3
YWHAZ	pp	TNFAIP3
FURIN	pp	TNFSF13
RNF11	pp	TNIP1
RELA	pp	TNIP2
SFRS5	pp	TNK2
WWOX	pp	TNK2

FNBP1	pp	TNKS
MCL1	pp	TNKS
POT1	pp	TNKS
TNKS1BP1	pp	TNKS
TNKS2	pp	TNKS
VPS33A	pp	TNKS
TNKS2	pp	TNKS1BP1
LNPEP	pp	TNKS2
TERF1	pp	TNKS2
CA12	pp	TOE1
SUMO1	pp	TOE1
TOE1	pp	TOE1
ATF3	pp	TP53
BCL2L1	pp	TP53
CDC42	pp	TP53
CDK2	pp	TP53
CDK5	pp	TP53
CEBPZ	pp	TP53
CHUK	pp	TP53
COPS4	pp	TP53
COPS5	pp	TP53
CREBBP	pp	TP53
CSNK1D	pp	TP53
CSNK2A1	pp	TP53
CSNK2B	pp	TP53
DDX5	pp	TP53
GSK3B	pp	TP53
HABP4	pp	TP53
HIF1A	pp	TP53
HNF4A	pp	TP53
KAT2B	pp	TP53
KPNB1	pp	TP53
MAPK1	pp	TP53
MAPK3	pp	TP53
MAPK8	pp	TP53
MED1	pp	TP53
MNAT1	pp	TP53
PIAS4	pp	TP53
PPP2CA	pp	TP53
PRKCA	pp	TP53
PRKDC	pp	TP53
PRMT1	pp	TP53
PTGS2	pp	TP53
PTK2	pp	TP53

SIN3A	pp	TP53
SIRT1	pp	TP53
SUMO1	pp	TP53
TBP	pp	TP53
THRB	pp	TP53
TK1	pp	TP53
TP53	pp	TP53
TP73	pp	TP53
TSG101	pp	TP53
UBE2I	pp	TP53
WWOX	pp	TP53
YWHAG	pp	TP53
YWHAZ	pp	TP53
ABL1	pp	TP73
CDK1	pp	TP73
CDK2	pp	TP73
CEBPZ	pp	TP73
SMAD2	pp	TP73
TP73	pp	TP73
WWOX	pp	TP73
BIRC2	pp	TRAF1
BIRC3	pp	TRAF1
CASP10	pp	TRAF1
CASP3	pp	TRAF1
RIPK1	pp	TRAF1
TICAM1	pp	TRAF1
TRAIP	pp	TRAF1
ATXN1	pp	TRAF2
BCL10	pp	TRAF2
CYLD	pp	TRAF2
GSTP1	pp	TRAF2
IKBKB	pp	TRAF2
MAP3K5	pp	TRAF2
PKN1	pp	TRAF2
RIPK1	pp	TRAF2
SIAH2	pp	TRAF2
TRAF1	pp	TRAF2
TRAF2	pp	TRAF2
TRAIP	pp	TRAF2
CASP3	pp	TRAF3
RIPK1	pp	TRAF3
TRAF3	pp	TRAF3
TRAF3IP3	pp	TRAF3
ABL1	pp	TRAF6

CAV1	pp	TRAF6
MAP2K1	pp	TRAF6
MAP2K6	pp	TRAF6
MAP3K5	pp	TRAF6
MAP3K7	pp	TRAF6
PSMC2	pp	TRAF6
PSMC3	pp	TRAF6
SIGIRR	pp	TRAF6
TAX1BP1	pp	TRAF6
TGFBR1	pp	TRAF6
TICAM1	pp	TRAF6
TRAF2	pp	TRAF6
TRAF6	pp	TRAF6
CYLD	pp	TRAF7
TRAF6	pp	TRAF7
TRAF7	pp	TRAF7
CYLD	pp	TRAIP
PRSS23	pp	TRAPPC4
GMEB1	pp	TRIM63
TRIM63	pp	TRIM63
UBE2I	pp	TRIM63
CDK1	pp	TSC1
NF2	pp	TSC1
RDX	pp	TSC1
YWHAG	pp	TSC1
TSC22D3	pp	TSC22D3
CDKN1A	pp	TTLL5
EGFR	pp	TUBA4A
ENO2	pp	TUBA4A
HSPH1	pp	TUBA4A
RAC1	pp	TUBA4A
SIAH1	pp	TUBA4A
TAOK2	pp	TUBA4A
YWHAG	pp	TUBA4A
BMPR2	pp	TUBB
ESR1	pp	TUBB
TAOK2	pp	TUBB
YWHAB	pp	TUBB
YWHAG	pp	TUBB
YWHAZ	pp	TUBB
CDK1	pp	UBA1
HSPH1	pp	UBA1
SEMA3F	pp	UBA1
ATXN1	pp	UBAP2L

IGF1R	pp	UBC
PPARGC1A	pp	UBC
RNF11	pp	UBC
YWHAB	pp	UBC
YWHAG	pp	UBC
YWHAZ	pp	UBC
TGFBR1	pp	UBD
ENO2	pp	UBE2C
UBE2C	pp	UBE2C
ATXN1	pp	UBE2I
ELK1	pp	UBE2I
ESR1	pp	UBE2I
GMEB1	pp	UBE2I
HABP4	pp	UBE2I
HNF4A	pp	UBE2I
HNRNPK	pp	UBE2I
LMNA	pp	UBE2I
SKIL	pp	UBE2I
SOX10	pp	UBE2I
UBE2I	pp	UBE2I
KITLG	pp	UBE2V1
SUMO4	pp	UBE2V1
HK2	pp	UBQLN1
P4HB	pp	UBQLN1
UBQLN1	pp	UBQLN1
HK2	pp	UBQLN4
PTN	pp	UBQLN4
RNF11	pp	UBQLN4
RUNX1T1	pp	UBQLN4
RXRA	pp	UBQLN4
SPP1	pp	UBQLN4
TRAF2	pp	UBQLN4
UBQLN1	pp	UBQLN4
UBQLN4	pp	UBQLN4
YWHAQ	pp	UBQLN4
BAT5	pp	UPF1
NDRG1	pp	UPF1
PPARGC1A	pp	USF2
USF2	pp	USF2
QKI	pp	USH1C
USH1C	pp	USH1C
YWHAG	pp	USP37
CTSG	pp	VCAM1
MSN	pp	VCAM1

VCAM1	pp	VCAM1
NDRG1	pp	VCP
SUMO4	pp	VCP
VCP	pp	VCP
NRP1	pp	VEGFA
NRP2	pp	VEGFA
SEMA3F	pp	VEGFA
VEGFA	pp	VEGFA
EPAS1	pp	VHL
FLNA	pp	VHL
HIF1A	pp	VHL
HIF3A	pp	VHL
SP1	pp	VHL
CASP3	pp	VIM
CASP9	pp	VIM
CDH5	pp	VIM
CDK1	pp	VIM
DCTN1	pp	VIM
PIAS4	pp	VIM
PKN1	pp	VIM
PPHLN1	pp	VIM
ROCK1	pp	VIM
SMAD3	pp	VIM
VIM	pp	VIM
YWHAE	pp	VIM
YWHAZ	pp	VIM
TRAF2	pp	WDR65
EGLN3	pp	WDR83
AKT1	pp	WNK1
YWHAG	pp	WNK1
XBP1	pp	XBP1
AKT1	pp	XIAP
CASP3	pp	XIAP
CASP9	pp	XIAP
DIABLO	pp	XIAP
MAP3K7	pp	XIAP
TRAF6	pp	XIAP
XIAP	pp	XIAP
AR	pp	XRCC5
NDRG1	pp	XRCC5
RBM14	pp	XRCC5
SGOL1	pp	XRCC5
XRCC5	pp	XRCC5
ABL1	pp	XRCC6

AR	pp	XRCC6
CCNA1	pp	XRCC6
MAPK8	pp	XRCC6
PARP1	pp	XRCC6
PCNA	pp	XRCC6
PRKDC	pp	XRCC6
SGOL1	pp	XRCC6
TP53	pp	XRCC6
XRCC5	pp	XRCC6
ABL1	pp	YWHAB
BAD	pp	YWHAB
CDK14	pp	YWHAB
IKBKB	pp	YWHAB
PRKCD	pp	YWHAB
PRKCZ	pp	YWHAB
RPS6KA1	pp	YWHAB
TSC1	pp	YWHAB
YWHAB	pp	YWHAB
YWHAG	pp	YWHAB
ABL1	pp	YWHAE
CASP3	pp	YWHAE
CDK14	pp	YWHAE
HSF1	pp	YWHAE
MAP3K2	pp	YWHAE
NCOR2	pp	YWHAE
PRKCG	pp	YWHAE
TSC1	pp	YWHAE
YWHAB	pp	YWHAE
YWHAZ	pp	YWHAE
ABL1	pp	YWHAG
BAD	pp	YWHAG
CRTC2	pp	YWHAG
EEF1G	pp	YWHAG
KIF5B	pp	YWHAG
MAP3K2	pp	YWHAG
PAK4	pp	YWHAG
YWHAE	pp	YWHAG
YWHAG	pp	YWHAG
ABL1	pp	YWHAH
BAD	pp	YWHAH
CREBBP	pp	YWHAH
ESR1	pp	YWHAH
ESR2	pp	YWHAH
KCNH2	pp	YWHAH

MAP3K5	pp	YWHAH
MED1	pp	YWHAH
NCOA1	pp	YWHAH
NR3C1	pp	YWHAH
RAF1	pp	YWHAH
THRA	pp	YWHAH
TNFAIP3	pp	YWHAH
TSC1	pp	YWHAH
YWHAE	pp	YWHAH
YWHAG	pp	YWHAH
ABL1	pp	YWHAQ
BAD	pp	YWHAQ
CABIN1	pp	YWHAQ
CDK14	pp	YWHAQ
CSNK2A1	pp	YWHAQ
KIF5B	pp	YWHAQ
MAP3K5	pp	YWHAQ
MTNR1B	pp	YWHAQ
PDK1	pp	YWHAQ
PRKD1	pp	YWHAQ
TSC1	pp	YWHAQ
YWHAG	pp	YWHAQ
YWHAQ	pp	YWHAQ
ABL1	pp	YWHAZ
AKT1	pp	YWHAZ
ATXN1	pp	YWHAZ
BAD	pp	YWHAZ
CRTC2	pp	YWHAZ
LIMK1	pp	YWHAZ
MAP3K2	pp	YWHAZ
MAP3K5	pp	YWHAZ
MAPK8	pp	YWHAZ
PRKCD	pp	YWHAZ
TSC1	pp	YWHAZ
XRCC6	pp	YWHAZ
YWHAZ	pp	YWHAZ
HDAC2	pp	YY1
CREBBP	pp	ZBTB17
ESR1	pp	ZBTB17
ESR2	pp	ZBTB17
HK2	pp	ZBTB17
PGR	pp	ZBTB17
RIPK1	pp	ZFAND5
TNFAIP3	pp	ZFAND5

TRAF6	pp	ZFAND5
ZFAND5	pp	ZFAND5
NDRG1	pp	ZNF155
COIL	pp	ZNF277

Supplementary Material IV. A new DEG PPI network derived from STRING dataset.

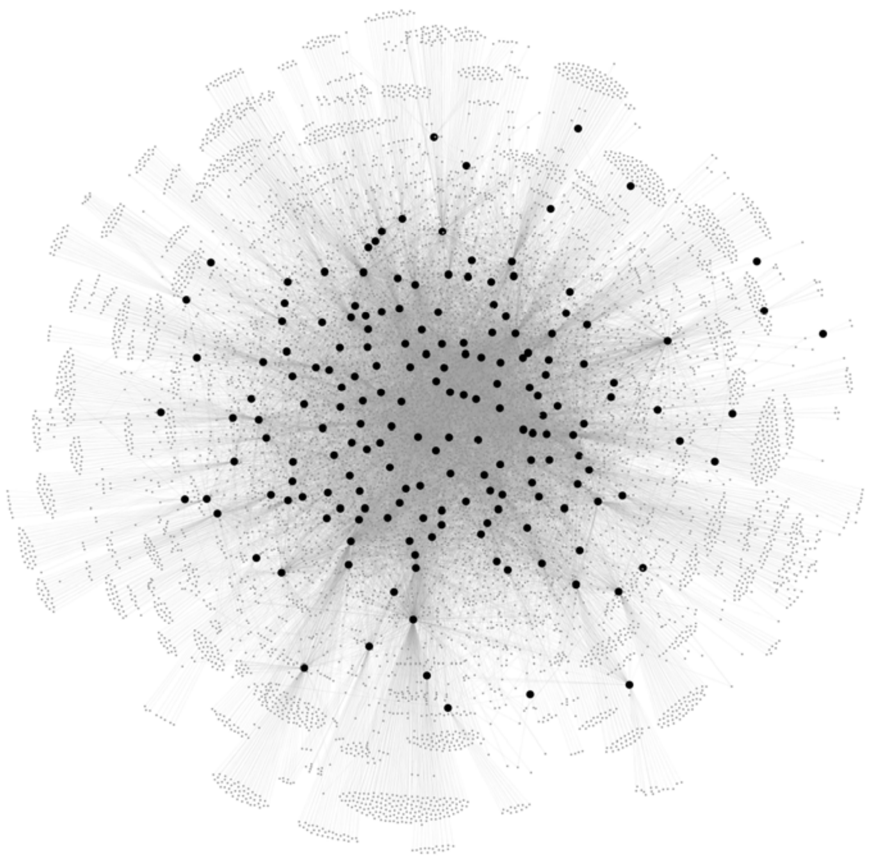

Supplementary Material IV. A new DEG PPI network derived from STRING dataset, which contain 8362 nodes (179 DEGs) and 23117 edges. The black nodes present the DEGs, the white small nodes present their interacting proteins.

Supplementary Material V. The distributions of node degree of new DEG network derived from STRING dataset.

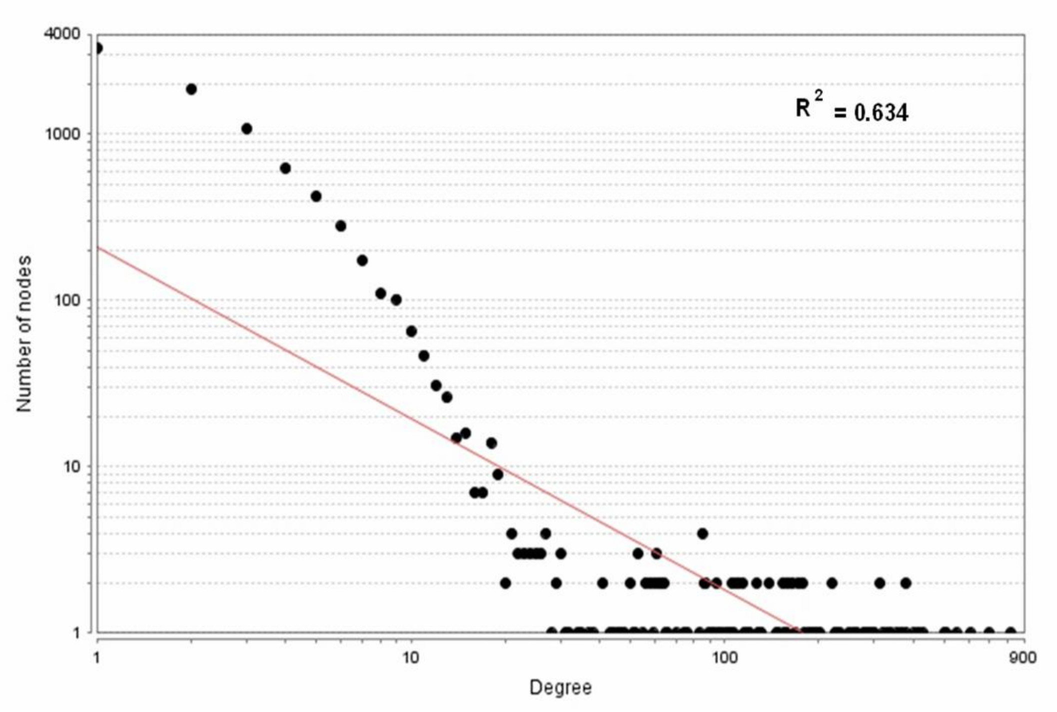

Supplementary Material V. Power law distribution of node degree.

Supplementary Material IV. Topological parameters of the total DEG PPI sub-network derived from STRING

PPI sub-network	$y = \beta x^a$	R^2	correlation	clustering coefficient	network centralization	network density
Total DEGs	$y = 209.57x^{-1.033}$	0.634	0.978	0.106	0.097	0.001
